# Adverse Events Following Varicella-Containing Vaccination in Healthy Children: A Systematic Review and Meta-Analysis

**DOI:** 10.3390/vaccines14100910

**Published:** 2026-10-20

**Authors:** Haoyang Hu, Yeran Jiaerken, Chaofan Wang, Hai Fang

**Affiliations:** 1 School of Public Health, Peking University, Beijing 100191, China; 2 China Center for Health Development Studies, Peking University, Beijing 100191, China

**Keywords:** varicella vaccine, MMRV vaccine, vaccine safety, adverse events, children, randomized controlled trials, meta-analysis

## Abstract

**Background:** Varicella-containing vaccines have substantially reduced the burden of varicella worldwide; however, comprehensive evidence on adverse events following vaccination in children remains important for vaccine safety monitoring, vaccine confidence and immunization policy. We aimed to systematically evaluate the incidence of adverse events following licensed varicella-containing vaccination in healthy children. **Methods:** We conducted a systematic review and meta-analysis of randomized controlled trials (RCTs) evaluating monovalent varicella vaccines or measles–mumps–rubella–varicella (MMRV) vaccines in healthy children aged ≥12 months and <12 years. Eight databases were searched from 1 January 2000 to 17 July 2026. Studies using active safety monitoring and reporting extractable adverse event counts with corresponding denominators were included. Pooled incidence proportions among vaccinated children were estimated using generalized linear mixed models with random effects. Subgroup analyses were performed by vaccine type, vaccination dose, and World Health Organization region. Risk of bias was assessed using the Cochrane Risk of Bias 2 tool, and certainty of evidence was evaluated using the GRADE approach. **Results:** Forty-three RCTs involving 31,915 vaccinated children were included. Fever was the most frequently reported common adverse event, with a pooled incidence of 11.58% (95% confidence interval [CI], 6.79–19.07%). Pooled incidences were 2.74% (95% CI, 1.10–6.67%) for redness/erythema, 2.19% (95% CI, 0.93–5.06%) for pain, and 0.82% (95% CI, 0.33–1.98%) for swelling. Compared with monovalent varicella vaccines, MMRV vaccines showed higher pooled incidences of fever (43.44% vs. 5.29%), redness/erythema (16.50% vs. 0.69%), pain (12.27% vs. 0.91%), and swelling (7.02% vs. 0.30%); however, these estimates were based on between-study comparisons and should not be interpreted as causal effects of vaccine type. Rare serious adverse events were uncommon, including febrile seizures (0.0989%) and anaphylaxis (0.0343%), with no observed cases of encephalitis. **Conclusions:** Varicella-containing vaccines were generally well tolerated in children, with most observed adverse events being uncommon and rare serious adverse events infrequently reported. Continued safety surveillance remains important, particularly for rare serious adverse events.

## 1. Introduction

Varicella (chickenpox) is a highly contagious disease caused by varicella-zoster virus (VZV) and occurs worldwide, with the highest incidence traditionally observed among children in the absence of vaccination programs [1]. Although varicella is generally considered a mild childhood illness, it can cause clinically important complications, including secondary bacterial infections, pneumonia, neurological complications, hospitalization, and, rarely, death [2]. Beyond its direct clinical consequences, varicella can increase healthcare utilization, cause school absenteeism, impose caregiving burdens on families, and generate associated costs. The introduction of live attenuated varicella vaccines into routine childhood immunization programs has substantially reduced varicella incidence, hospitalizations, and severe complications in countries with established vaccination programs [3]. Varicella-containing vaccines, including monovalent varicella vaccines and combined measles–mumps–rubella–varicella (MMRV) vaccines, are now widely used globally as important components of childhood immunization strategies [1].

The safety profile of varicella-containing vaccines is a critical consideration for maintaining public confidence and optimizing immunization policies [4]. As these vaccines are administered primarily to healthy children, even uncommon adverse events may influence vaccine acceptance and program implementation. Previous clinical trials and post-licensure surveillance studies have generally demonstrated that varicella-containing vaccines are well tolerated, with most reported adverse events being mild and transient, such as fever and injection-site reactions [2,5,6]. Rare serious adverse events, including febrile seizures, anaphylaxis, immune thrombocytopenic purpura (ITP), and encephalitis, have also been reported, although their occurrence appears to be uncommon.

However, interpretation of the existing safety evidence remains challenging because individual studies vary substantially in vaccine formulations, vaccination schedules, participant characteristics, safety-monitoring methods, outcome definitions, and observation periods. In particular, comparisons between monovalent varicella vaccines and MMRV vaccines have generated interest because some studies have suggested differences in fever and other adverse events after vaccination [7,8]. MMRV combines measles, mumps, rubella, and varicella antigens in a single injection and may simplify the vaccination schedule by reducing the number of injections, whereas separate administration of these vaccines requires additional injections. However, a comparison of adverse event incidence between MMRV and monovalent varicella vaccine alone does not establish whether MMRV differs in safety from measles–mumps–rubella (MMR) vaccine plus separately administered monovalent varicella vaccine. Furthermore, individual randomized controlled trials are often insufficiently powered to evaluate rare serious adverse events.

Previous systematic reviews have summarized the safety of varicella-containing vaccines; however, several limitations remain [8,9,10]. Some reviews included observational studies together with randomized trials, potentially introducing heterogeneity in study design and outcome ascertainment. Others focused on specific vaccine products or particular adverse events, limiting the comprehensiveness of safety evaluation. In addition, differences in adverse event definitions and inconsistent reporting of denominators have made it difficult to estimate pooled incidences of individual adverse events across studies. A comprehensive synthesis restricted to randomized controlled trials with active safety monitoring and extractable event counts and denominators is therefore needed.

In this systematic review and meta-analysis, we aimed to comprehensively evaluate the incidence of adverse events following licensed varicella-containing vaccination among healthy children based on randomized controlled trial evidence. We estimated the pooled incidences of common adverse events, examined differences according to vaccine type and vaccination dose, explored potential regional variation, and summarized rare serious adverse events. These analyses describe adverse events observed following vaccination and do not estimate vaccine-attributable risks. In particular, comparisons by vaccine type were based primarily on between-study subgroup analyses and did not directly evaluate MMRV versus MMR plus separately administered monovalent varicella vaccine. By integrating evidence from randomized trials worldwide, this study provides an updated assessment of the frequency of adverse events observed following varicella-containing vaccination and contributes to the evidence base for vaccine safety monitoring and childhood immunization programs.

## 2. Methods

### 2.1. Study Design and Reporting

This systematic review and meta-analysis was conducted to evaluate the incidence of adverse events following varicella-containing vaccination in healthy children. The review protocol was developed prospectively by the study team before data extraction and statistical analysis, with predefined eligibility criteria, outcomes, subgroup analyses, statistical methods, and sensitivity analyses. This study was conducted and reported according to the Preferred Reporting Items for Systematic Reviews and Meta-Analyses (PRISMA) 2020 statement. Because the primary analysis pooled incidence proportions within vaccinated study groups, the resulting estimates were interpreted as descriptive measures of adverse events observed following vaccination rather than comparative or causal measures of vaccine-attributable risk.

### 2.2. Literature Search Strategy

We systematically searched eight electronic databases, including PubMed, Embase, the Cochrane Central Register of Controlled Trials (CENTRAL), Web of Science, Scopus, China National Knowledge Infrastructure (CNKI), Wanfang Data, and the VIP Chinese Science Journals Database, from 1 January 2000 to 17 July 2026 inclusive.

The search strategy combined controlled vocabulary and free-text terms using two main concept blocks: varicella vaccination and vaccine safety. The vaccination-related terms included “varicella vaccine”, “varicella vaccin*”, “chickenpox vaccine”, “varicella zoster virus”, “VZV”, “MMRV”, and “measles mumps rubella varicella”. The safety-related terms included “Drug-Related Side Effects and Adverse Reactions”, “adverse event*”, “adverse reaction*”, “adverse event following immunization”, “post-vaccination reaction*”, “safety”, “side effect*”, “AEFI”, “serious adverse event*”, “SAE”, “local reaction*”, and “systemic reaction*”. The two concept blocks were combined using the Boolean operator AND. Equivalent Chinese terms were used to search the Chinese databases. The complete search strategies for each database are provided in Supplementary Materials Section S1.3.

### 2.3. Eligibility Criteria

Studies were eligible if they met the following criteria:

(1) **Population:** healthy children aged ≥ 12 months and <12 years at vaccination. Studies including broader age ranges were eligible only when data for the target population could be separately extracted.
(2) **Intervention:** licensed monovalent live attenuated varicella vaccines or measles–mumps–rubella–varicella (MMRV) vaccines.
(3) **Study design:** individual-level or cluster randomized controlled trials.
(4) **Safety assessment:** studies using prospectively defined active safety-monitoring methods, including diary cards, caregiver-recorded symptom reports, scheduled visits, or active telephone follow-up.
(5) **Outcome reporting:** studies reporting at least one predefined adverse event with available event counts and corresponding safety-analysis denominators from the same study group, vaccination dose, and observation period.

Studies were excluded if they involved non-randomized designs, special populations without separable data for healthy children, unlicensed or investigational vaccines, or studies in which adverse events attributable to varicella-containing vaccines could not be distinguished from concomitant vaccinations. Studies without extractable numerators and denominators, duplicate reports, reviews, protocols, case reports, and conference abstracts without sufficient data were also excluded.

### 2.4. Study Selection and Data Extraction

Records retrieved from the eight databases were exported and deduplicated before screening. Two reviewers independently screened titles and abstracts, followed by full-text assessment of potentially eligible reports. Disagreements were resolved through discussion or consultation with a third reviewer.

The same reviewers independently extracted data using a standardized extraction form and cross-checked the extracted information against the full-text reports. Multiple reports from the same trial were linked to avoid double-counting participants. The following information was extracted: study characteristics, publication year, study country or region, vaccine type, vaccination dose, safety-monitoring method, observation period, adverse event definitions, number of participants experiencing each adverse event, and corresponding safety-analysis denominators.

For multi-arm trials, eligible varicella-containing vaccine groups were extracted separately. A study group was included in the single-arm synthesis only when both the adverse event count and the corresponding safety-analysis denominator were available for the same group, vaccination dose, and observation period. Comparator groups were documented, including the availability of usable comparator data, but were not included in the single-arm synthesis. Missing values or unclear codes were not treated as zero events unless the source report explicitly stated that no events had occurred. Different adverse event categories were not summed because individual symptoms could occur in the same participant. Additional data-verification and handling procedures are described in Supplementary Materials Sections S4.2, S4.4 and S4.5.

### 2.5. Outcomes

The outcomes assessed in this study included adverse events following varicella vaccination, including injection-site pain, erythema, swelling, injection-site rash, fever, generalized rash, gastrointestinal symptoms, fatigue, irritability, and cough. Rare serious adverse events of clinical interest included febrile seizures, anaphylaxis, immune thrombocytopenic purpura (ITP), and encephalitis.

The prespecified observation windows were 0–7 days after vaccination for injection-site adverse events and 0–14 days for fever and other common systemic adverse events. For serious adverse events, event counts were extracted over the available follow-up period, which was generally days 0–42 after vaccination. This extraction period reflected the follow-up information reported in the source trials and was not intended to represent a biologically equivalent risk window for the different serious adverse events. When multiple definitions or observation windows were reported for the same outcome, data meeting the prespecified criteria were preferentially extracted.

Because many included studies did not report the proportion of participants experiencing at least one adverse event (overall adverse event rate), and individual adverse events could occur in the same participant, an overall adverse event rate could not be consistently calculated across studies. Therefore, the analysis focused on prespecified individual adverse events rather than an overall adverse event rate.

### 2.6. Risk of Bias and Certainty of Evidence

The risk of bias of included randomized controlled trials was assessed independently by two reviewers using the Cochrane Risk of Bias 2 (RoB 2) tool, which evaluates five domains: bias arising from the randomization process, bias due to deviations from intended interventions, bias due to missing outcome data, bias in outcome measurement, and bias in selection of the reported result. Each domain and the overall risk of bias were classified as low risk, some concerns, or high risk according to the RoB 2 algorithm. Disagreements were resolved through discussion or consultation with a third reviewer. The risk-of-bias assessments were recorded at the trial level based on the safety outcomes considered during study eligibility assessment; they were not conducted separately for each individual safety outcome. Accordingly, these assessments should not be interpreted as outcome-specific judgments for individual adverse events.

The certainty of evidence for the outcomes of interest was assessed using the GRADE (Grading of Recommendations Assessment, Development and Evaluation) approach. Evidence from randomized controlled trials was initially rated as high certainty and was downgraded, when appropriate, for risk of bias, inconsistency, indirectness, imprecision, and publication bias. The certainty of evidence was assessed separately for the overall pooled estimates and vaccine-type-specific estimates. Detailed criteria for risk-of-bias judgments and GRADE downgrading are provided in Supplementary Materials Sections S6.1 and S6.3.

### 2.7. Statistical Analysis

All statistical analyses were performed using R version 4.6.1 (R Foundation for Statistical Computing, Vienna, Austria), with the meta package. For common adverse events, pooled incidence proportions and corresponding 95% confidence intervals (CIs) among vaccinated study groups were estimated using generalized linear mixed models (GLMMs) with a logit link and a random-intercept model. The pooled estimates were model-based summaries of study-specific incidence proportions on the logit scale and were back-transformed to the incidence scale; they were therefore not equivalent to crude proportions calculated across all participants. These pooled estimates were interpreted as descriptive measures of the frequency of adverse events observed following vaccination rather than measures of vaccine-attributable risk. Statistical heterogeneity was assessed using the I^2^ statistic and between-study variance (τ^2^), and 95% prediction intervals were calculated where appropriate.

Prespecified subgroup analyses were conducted according to vaccine type (monovalent varicella vaccine vs. MMRV) and vaccination dose (first dose and second dose). Exploratory subgroup analyses were also conducted according to World Health Organization (WHO) region, including the African Region, Region of the Americas, South-East Asia Region, European Region, Eastern Mediterranean Region, and Western Pacific Region. Studies conducted across two or more WHO regions were classified separately as multi-region studies. Differences between subgroups were assessed using the test for subgroup differences under the random-effects model. Because these subgroup analyses primarily compared study groups rather than randomized comparisons within the same trials, differences between subgroups were interpreted as descriptive associations rather than causal effects. In particular, the MMRV and monovalent varicella vaccine groups were not considered equivalent vaccine strategies, and this analysis did not compare MMRV with MMR plus separately administered monovalent varicella vaccine.

WHO-region analyses were restricted to common adverse events with sufficient data. When only one study group was available within a region, the observed incidence and exact 95% CI based on the Clopper–Pearson method were reported without pooling. Regions without eligible data were reported as having insufficient evidence. Rare serious adverse events, including febrile seizures and encephalitis, were summarized using the observed incidence and exact 95% CIs and were not subjected to regional subgroup analyses.

Trials included in the quantitative synthesis are cited in the Results [11,12,13,14,15,16,17,18,19,20,21,22,23,24,25,26,27,28,29,30,31,32,33,34,35,36,37,38,39,40,41,42,43,44,45,46,47,48,49,50,51,52,53]. Sensitivity analyses were conducted by excluding studies with children less than 12 months, and, for fever, by excluding studies judged to have an overall high risk of bias. When at least 10 study groups were available for an outcome, funnel plots were examined and Egger’s linear regression test was used to explore potential small-study effects. Given the characteristics of single-proportion meta-analysis, including substantial heterogeneity and the presence of rare or zero-event outcomes, asymmetry was not interpreted as direct evidence of publication bias.

For each outcome, event counts and corresponding safety-analysis denominators were analyzed separately according to the prespecified observation window and vaccination dose. When first- and second-dose data were available from the same participants, they were analyzed separately and were not combined across doses.

All statistical tests were two-sided, and *p* < 0.05 was considered statistically significant. Detailed procedures for handling rare events, zero events, WHO-region classification, within-study data aggregation, and sensitivity analyses are provided in Supplementary Materials Sections S5.6–S5.8, S7.1 and S7.2.

## 3. Results

### 3.1. Literature Search and Study Selection

**Figure 1:**
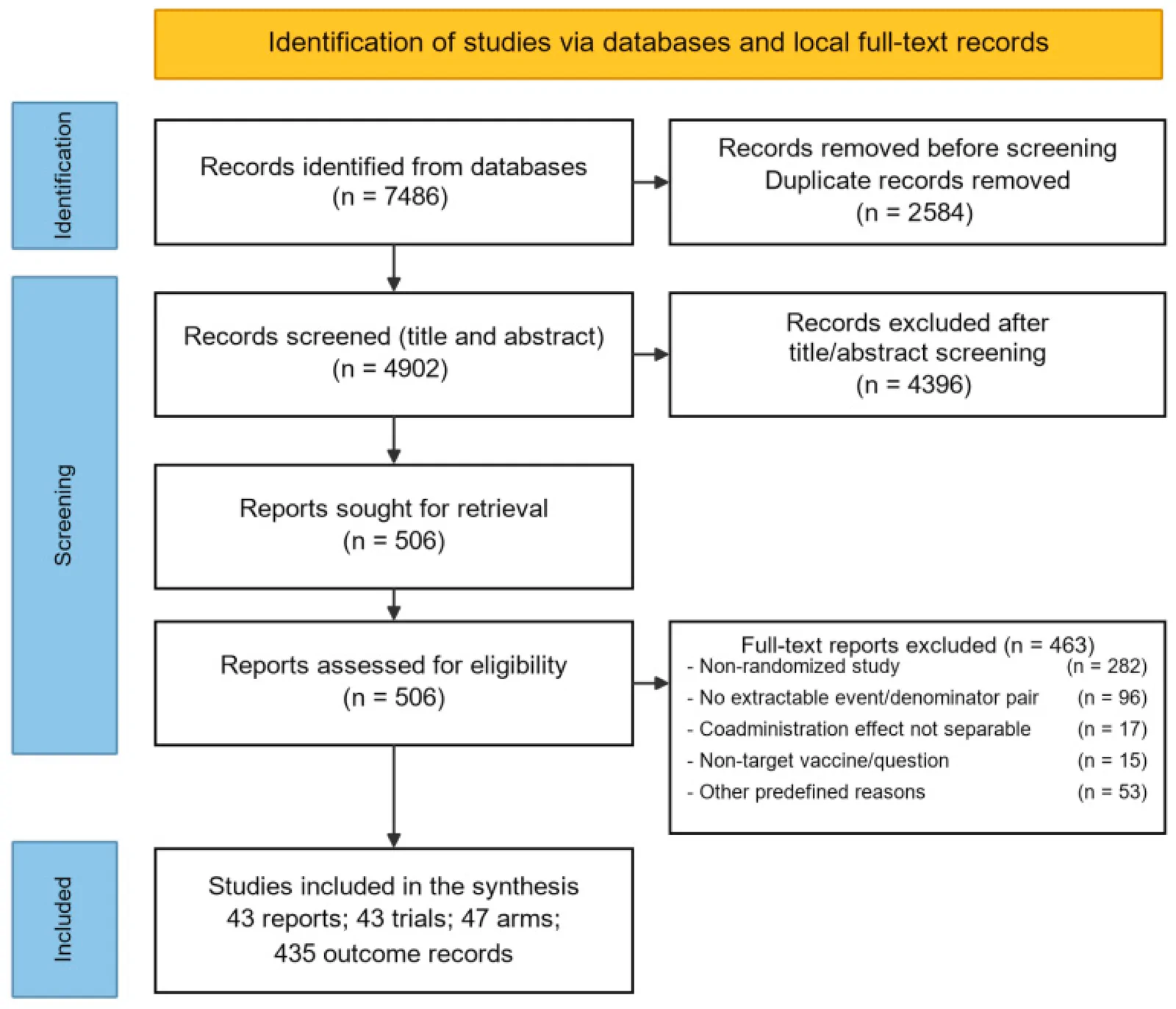
PRISMA 2020 flow diagram of study selection.

The initial search identified 7486 records. After removal of 2584 duplicates, 4902 records remained, of which 4396 were excluded based on title and abstract screening. Full texts were sought for 506 records. Following full-text assessment, 463 reports were excluded, leaving 43 reports corresponding to 43 independent randomized trials for inclusion in the systematic review and meta-analysis (Figure 1).

The main reasons for exclusion at the full-text stage were non-randomized study designs (n = 282), inability to extract event counts and corresponding denominators for the same study group (n = 96), inability to distinguish adverse events attributable to varicella-containing vaccination from those associated with concomitant vaccinations (n = 17), and non-target vaccines or research questions (n = 15).

### 3.2. Characteristics of Included Studies

The 43 included randomized trials were published between 2002 and 2025 and were conducted in 24 countries. These trials contributed 47 eligible study groups and included a total of 31,915 vaccinated children with verifiable safety-analysis denominators. This total represents the sum of the safety-analysis populations across eligible vaccination groups; different vaccination groups within the same trial were counted as separate groups with non-overlapping participants, and non-target vaccine control groups were excluded.

According to the location where the trials were conducted, 18 trials were classified in the WHO Western Pacific Region (WPR), 9 in the European Region (EUR), 6 in the Region of the Americas (AMR), 4 in the South-East Asia Region (SEAR), and 6 as cross-region studies. No eligible trials were identified from the African Region (AFR) or Eastern Mediterranean Region (EMR). The included studies varied in vaccine products, vaccination schedules, and active safety-monitoring procedures (Table 1).

All included trials used active safety monitoring, except for three trials in which safety monitoring was classified as mixed. Monitoring approaches included prospectively defined methods such as diary cards, caregiver-recorded symptoms, scheduled visits, and active follow-up. The vaccines included licensed monovalent live attenuated varicella vaccines and measles–mumps–rubella–varicella (MMRV) vaccines, with substantial variation in vaccine products and formulations across trials.

### 3.3. Incidence of Adverse Events

Because different adverse events could occur in the same vaccinated child, event counts for individual symptoms were not summed to derive an overall adverse event rate. Among the 43 included reports, 11 provided identifiable information on a broadly defined “any adverse event” outcome. Of these, 6 reported exact numbers of participants experiencing at least one adverse event together with the corresponding safety-analysis denominators, whereas 5 reported only percentages. After prespecified within-study aggregation of independent vaccination groups containing varicella vaccine, study-level descriptive rates ranged from approximately 28.0% to 87.3%. When a trial included more than one vaccine dose, results were presented separately by dose where applicable. Most of these reports used observation periods of approximately 42 days after vaccination; minor differences included 42 versus 43 days and day 0–42 versus day 1–42. Given substantial differences in adverse event definitions, vaccine products, vaccination doses, and safety-analysis populations, these rates were not quantitatively pooled and are presented descriptively in Supplementary Materials Section S9.1. They should not be interpreted as evidence of differences in safety according to vaccine type, vaccination dose, or geographic region.

Fever was reported by 45 study groups, with 5261 events among 31,472 vaccinated children (Table 2). The pooled incidence was 11.58% (95% CI, 6.79–19.07%) using a random-effects generalized linear mixed model (GLMM). Between-study heterogeneity was extreme (I^2^ = 98.8%; τ^2^ = 3.9000), and the 95% prediction interval ranged from 0.23% to 88.00% (Supplementary Materials Section S9.2), indicating substantial variation in the observed incidence across study settings and limiting the interpretation and generalizability of the pooled estimate.

Pain, redness/erythema, and swelling were reported by 39, 36, and 40 study groups, respectively. The pooled incidences were 2.19% (95% CI, 0.93–5.06%; I^2^ = 97.0%), 2.74% (95% CI, 1.10–6.67%; I^2^ = 96.2%), and 0.82% (95% CI, 0.33–1.98%; I^2^ = 90.9%), respectively. All three injection-site adverse events showed substantial between-study heterogeneity, indicating that the pooled estimates should be interpreted as summaries of the observed evidence distribution rather than single incidence rates applicable across settings.

The pooled incidences of injection-site rash, generalized rash, gastrointestinal symptoms, fatigue, irritability, and cough were 0.048%, 0.407%, 0.058%, 0.010%, 0.039%, and 2.29%, respectively. Heterogeneity was absent for injection-site rash (I^2^ = 0%) but substantial for all other outcomes. For some low-frequency outcomes, the GLMM estimates differed substantially from the crude event proportions, particularly when between-study heterogeneity was high. For example, 840 gastrointestinal events were observed among 26,619 vaccinated children (3.16%), whereas the corresponding pooled GLMM incidence was 0.058%; similarly, 686 irritability events were observed among 15,278 vaccinated children (4.49%), compared with a pooled incidence of 0.039%. These pooled estimates represent model-based summaries of study-specific incidence proportions rather than the overall proportion of participants experiencing the event.

### 3.4. Subgroup Analysis by Vaccine Type

For the four common adverse events with sufficient data, observed pooled incidences were higher in the MMRV subgroup than in the monovalent varicella vaccine subgroup. Fever was reported in 14 MMRV study groups and 28 monovalent-varicella study groups, with pooled incidences of 43.44% (95% CI, 32.07–55.53%) and 5.29% (95% CI, 2.62–10.40%), respectively. Corresponding estimates for redness/erythema were 16.50% versus 0.69%, for pain 12.27% versus 0.91%, and for swelling 7.02% versus 0.30% (Supplementary Materials Section S9.3). These were descriptive between-study comparisons and may be confounded by age, product, dose, coadministration, monitoring definitions, and observation windows; they should not be interpreted as causal effects of vaccine type or as a comparison between MMRV and MMR plus separately administered monovalent varicella vaccine.

No statistically significant differences between vaccine-type subgroups were observed for injection-site rash, generalized rash, gastrointestinal symptoms, irritability, or cough (all *p* > 0.05). The confidence interval for fatigue was very wide, indicating an unstable model estimate.

### 3.5. Subgroup Analysis by Vaccination Dose

For fever, the pooled incidences were 10.46% (95% CI, 5.44–19.17%) after the first dose and 26.58% (95% CI, 13.55–45.55%) after the second dose (*p* = 0.043; Supplementary Materials Section S9.4). Dose-stratified estimates for other common adverse events are shown in Supplementary Materials Section S9.4. Estimates for some low-frequency outcomes were unstable and were not considered suitable for major clinical interpretation.

### 3.6. Exploratory Subgroup Analysis by WHO Region

Exploratory analyses were conducted by WHO region for common adverse events with sufficient data. For fever, 21, 9, 6, 4, and 5 study groups were included from the WPR, EUR, AMR, SEAR, and cross-region categories, respectively (Supplementary Materials Section S9.5). The corresponding pooled incidences were 4.38% (95% CI, 1.89–9.80%), 49.77% (95% CI, 38.50–61.06%), 13.00% (95% CI, 2.86–43.16%), 15.71% (95% CI, 3.73–47.32%), and 14.96% (95% CI, 12.52–17.77%), respectively. The difference between subgroups was statistically significant (Q = 58.71, df = 4, *p* < 0.001). Significant between-region differences were also observed for pain, redness/erythema, and swelling (*p* = 0.001, *p* = 0.016, and *p* < 0.001, respectively; Supplementary Materials Section S9.5). The distribution of WHO regions substantially overlapped with vaccine type. At the study-group level, WPR studies predominantly evaluated monovalent varicella vaccines, whereas EUR studies predominantly evaluated MMRV.

### 3.7. Rare Serious Adverse Events

The observed incidences of rare serious adverse events following vaccination, including febrile seizures, anaphylaxis, immune thrombocytopenic purpura (ITP), and encephalitis, are summarized in Supplementary Materials Section S9.6. These estimates were based on the available follow-up periods reported in the source trials, generally days 0–42 after vaccination, rather than event-specific biological risk windows. Febrile seizures were reported in 21 of 21,241 vaccinated children, corresponding to an observed incidence of 0.0989% (exact 95% CI, 0.0612–0.1511%). Eight cases of anaphylaxis were reported among 23,312 children, corresponding to an observed incidence of 0.0343% (exact 95% CI, 0.0148–0.0676%). One case of ITP occurred among 17,128 children, corresponding to an observed incidence of 0.00584% (exact 95% CI, 0.00015–0.0325%). No cases of encephalitis were observed among 17,128 vaccinated children.

### 3.8. Risk of Bias

**Figure 2:**
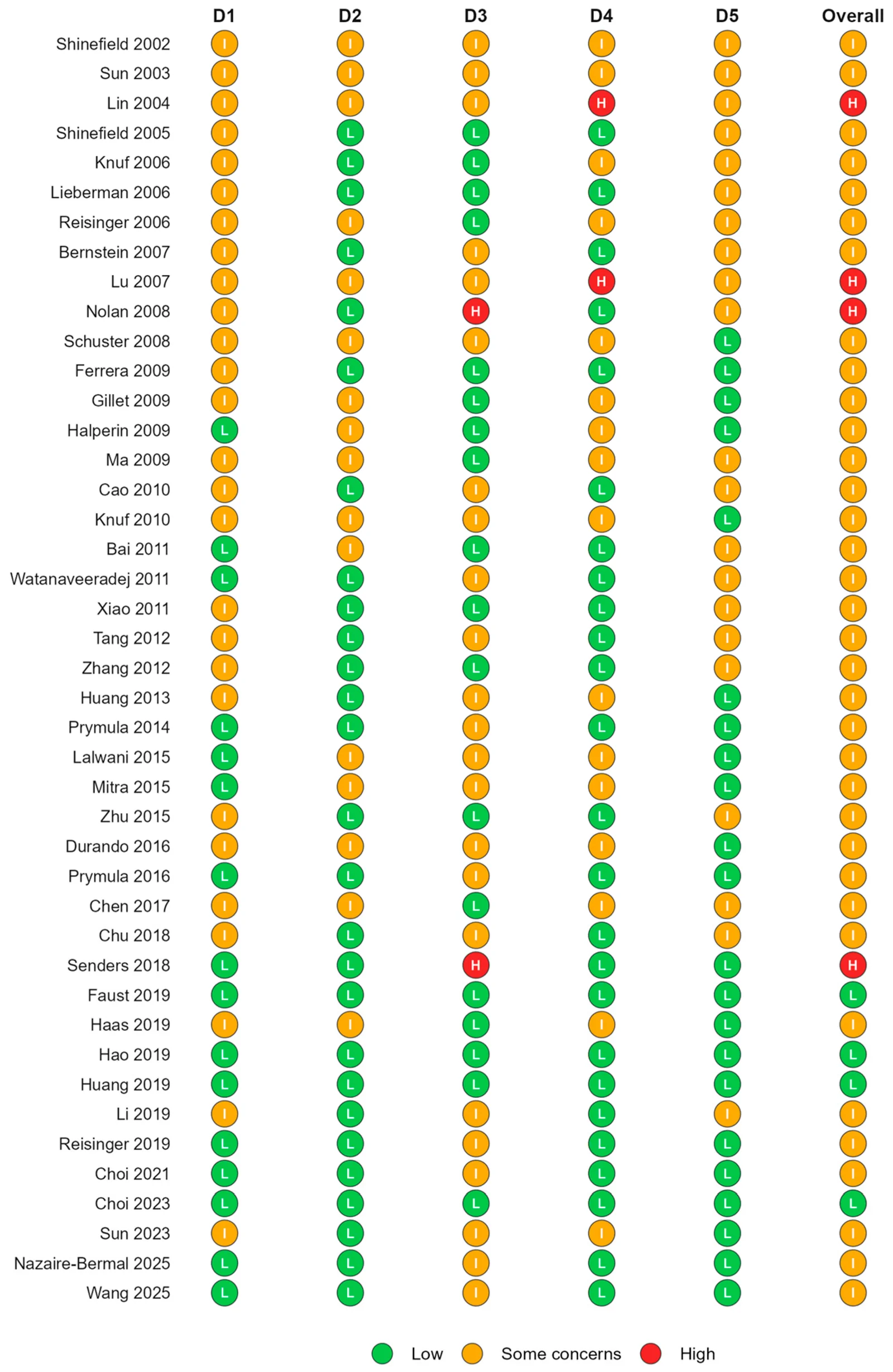
Risk-of-bias judgements for individual trials [11,12,13,14,15,16,17,18,19,20,21,22,23,24,25,26,27,28,29,30,31,32,33,34,35,36,37,38,39,40,41,42,43,44,45,46,47,48,49,50,51,52,53]. D1, bias arising from the randomization process; D2, bias due to deviations from intended interventions; D3, bias due to missing outcome data; D4, bias in outcome measurement; D5, bias in selection of the reported result.

Overall RoB 2 assessments indicated that 4 trials (9.3%) were at low risk of bias, 35 (81.4%) had some concerns, and 4 (9.3%) were at high risk of bias. These assessments were recorded at the trial level and should not be interpreted as outcome-specific risk-of-bias judgments for individual adverse events. For the randomization process, 16 trials (37.2%) were judged to be at low risk and 27 (62.8%) had some concerns (Figure 2). Two trials (4.7%) were judged to be at high risk of bias for missing outcome data, and another two trials (4.7%) were judged to be at high risk for outcome measurement. The four trials judged to have an overall high risk of bias were those by Lu et al. [19], Lin et al. [13], Senders et al. [42], and Nolan et al. [20] (Supplementary Materials Section S9.7).

### 3.9. Sensitivity Analyses

For fever, excluding the trial by Knuf et al. [27], which included a small proportion of children aged 11 months, resulted in 44 study groups comprising 5164 events among 31,306 children. The pooled incidence was 11.03% (95% CI, 6.43–18.30%), with I^2^ = 98.8%, τ^2^ = 3.8585, and a 95% prediction interval of 0.22–87.22%.

In a separate sensitivity analysis excluding the four trials judged to have an overall high risk of bias, 41 study groups comprising 5186 events among 30,254 children remained. The pooled fever incidence increased to 13.13% (95% CI, 7.65–21.63%), with I^2^ = 98.9%, τ^2^ = 3.7542, and a 95% prediction interval of 0.29–88.85%. Overall, exclusion of the age-boundary study had little effect on the pooled estimate, whereas exclusion of studies at high overall risk of bias resulted in a modest increase in the estimated incidence. This latter analysis should be interpreted as exploratory because the exclusion criteria were based on overall trial-level risk-of-bias judgments rather than an outcome-specific assessment for fever (Supplementary Materials Section S9.8).

Exploratory assessments of small-study effects were conducted for common adverse events with at least 10 study groups (Supplementary Materials Section S9.9). The funnel plot for fever was asymmetric. Egger’s test was statistically significant for fever, pain, redness/erythema, swelling, injection-site rash, generalized rash, gastrointestinal symptoms, fatigue, and irritability, but not for cough (*p* = 0.062). Given the single-proportion setting, substantial heterogeneity, and the presence of zero-event studies, these findings were not interpreted as evidence of publication bias.

### 3.10. Certainty of Evidence

The certainty of evidence varied according to the safety question evaluated (Table 3). The certainty of evidence for the overall pooled incidence of fever and the overall pooled incidences of pain, redness/erythema, and swelling was rated as low, primarily because of very substantial between-study inconsistency. For fever, the pooled estimate was 11.58% (95% CI, 6.79–19.07%), with I^2^ = 98.8% and a 95% prediction interval of 0.23–88.00%.

For vaccine-type-specific estimates, the certainty of evidence for fever was rated as moderate for both monovalent varicella vaccine and MMRV, with downgrading for residual inconsistency. The pooled fever incidences were 5.29% (95% CI, 2.62–10.40%) and 43.44% (95% CI, 32.07–55.53%), respectively. The certainty of evidence for vaccine-type-specific estimates of pain, redness/erythema, and swelling was also rated as moderate, with downgrading for inconsistency. These estimates represent between-study comparisons rather than direct randomized comparisons.

For febrile seizures, the certainty of evidence regarding whether the incidence was below 0.5% was rated as moderate because of imprecision, although the exact 95% CI for the observed incidence remained entirely below 0.5% (0.0612–0.1511%). For encephalitis, the certainty of evidence regarding whether the incidence was below 0.1% was rated as high because no cases were observed among 17,128 vaccinated children and the upper bound of the exact 95% CI was 0.0215%. Nevertheless, zero observed events do not imply zero underlying risk.

Overall, the pooled estimates should be interpreted as summary estimates of the observed evidence distribution rather than universal safety rates. Clinical interpretation should consider vaccine type, study context, prediction intervals, and sensitivity analyses. Vaccine-type subgroup findings should not be interpreted as causal effects.

## 4. Discussion

In this systematic review and meta-analysis of 43 randomized controlled trials involving 31,915 vaccinated children, we evaluated the incidence of adverse events following licensed varicella-containing vaccination in healthy children. Overall, most individual adverse events observed following vaccination were uncommon. Fever was the most frequently reported common adverse event, with a pooled incidence of 11.58%, followed by injection-site redness/erythema, pain, and swelling. In contrast, rare serious adverse events, including febrile seizures, anaphylaxis, immune thrombocytopenic purpura, and encephalitis, were uncommon. These findings provide descriptive evidence on the frequency of adverse events observed following varicella-containing vaccination and contribute to the evidence base for vaccine safety monitoring in childhood immunization programs. Our findings are generally consistent with previous clinical evidence showing that varicella vaccines are generally well tolerated in children [9,10,54]. Local reactions following vaccination were uncommon, with pooled incidences below 3% for pain and redness/erythema and below 1% for swelling. Systemic reactions other than fever, including generalized rash, gastrointestinal symptoms, fatigue, and irritability, occurred infrequently. Although fever was the most common adverse event identified, its incidence varied substantially across studies, as reflected by the wide prediction interval and high between-study heterogeneity [54]. This variability likely reflects differences in vaccine formulations, background characteristics of enrolled populations, vaccination schedules, definitions of adverse events, and intensity and timing of safety surveillance.

The comparison between vaccine types showed higher pooled incidences of fever and local reactions among children receiving MMRV vaccines than among those receiving monovalent varicella vaccines [55]. The pooled incidence of fever was 43.44% in the MMRV subgroup compared with 5.29% in the monovalent varicella vaccine subgroup. Similar patterns were observed for redness/erythema, pain, and swelling. These differences may be biologically plausible because MMRV vaccines contain additional live attenuated viral components and may elicit broader immune responses than monovalent varicella vaccines [56]. However, these findings should not be interpreted as causal effects of vaccine type or as evidence that MMRV is less safe than a separate MMR plus monovalent varicella vaccination strategy. The analyses were based on comparisons across independent study groups rather than direct randomized comparisons between vaccine types. Moreover, vaccine type was strongly correlated with geographic region, as MMRV trials were predominantly conducted in European and American settings, whereas monovalent varicella vaccine trials were mainly conducted in the Western Pacific Region. Therefore, observed differences may reflect variations in vaccine products, surveillance approaches, study populations, or regional clinical practices rather than vaccine composition alone. Importantly, the present analysis does not isolate the contribution of the varicella component within MMRV or directly compare MMRV with MMR plus separately administered monovalent varicella vaccine.

The dose-related analyses identified differences in fever and local reactions between first-dose and second-dose subgroups [9,57]. These findings require careful interpretation. First- and second-dose observations from the same participants may be correlated, and dose groups may also have differed systematically in vaccine type, age, vaccination schedule, and other study characteristics. Therefore, these analyses should be considered exploratory and should not be interpreted as evidence that subsequent doses inherently carry higher safety risks.

Exploratory analyses by WHO region demonstrated substantial geographic variation, particularly for fever and injection-site reactions [1]. However, regional differences overlapped considerably with vaccine-type distributions. For example, studies from the Western Pacific Region primarily evaluated monovalent varicella vaccines, whereas studies from European countries frequently evaluated MMRV vaccines. Differences in surveillance intensity, adverse event definitions, healthcare-seeking behavior, and reporting practices may also contribute to regional variation. Thus, geographic differences should not be interpreted as evidence of differential vaccine safety across regions.

The assessment of rare serious adverse events provides important information for vaccine safety monitoring [2]. Febrile seizures occurred in approximately 1 per 1000 vaccinated children, and the exact confidence interval remained below the clinically relevant threshold of 0.5%. Anaphylaxis was also rare, occurring in approximately 3 per 10,000 vaccinated children. Only isolated cases of immune thrombocytopenic purpura were identified, and no encephalitis cases were observed among more than 17,000 vaccinated children with available data. Although the absence of observed events does not indicate zero underlying risk, these data indicate that such serious adverse events were infrequently observed in the included study populations and monitoring periods.

A major strength of this study is the comprehensive inclusion of randomized controlled trials and the use of prospectively defined active safety monitoring as an eligibility criterion. Although the primary analysis did not use the randomized treatment comparisons, the RCT setting provided prospectively defined study populations and systematic safety monitoring, which supported the descriptive assessment of adverse event incidence. By restricting inclusion to studies with verifiable event counts and corresponding safety denominators, we aimed to minimize biases arising from incomplete reporting and inconsistent outcome definitions. Furthermore, we applied advanced statistical methods for single-proportion meta-analysis, conducted prespecified subgroup and sensitivity analyses, and assessed certainty of evidence using the GRADE framework.

Several limitations should be considered. First, the primary analysis pooled single-arm incidence proportions among vaccinated study groups and therefore did not directly estimate comparative risks between vaccinated and control groups or vaccine-attributable effects. The adverse events identified in these analyses represent events observed following vaccination and should not be interpreted as vaccine-attributable adverse reactions. The randomized comparisons available within some trials were consequently not fully exploited for causal inference. The pooled incidences should therefore be considered summaries of the observed evidence distribution rather than universal estimates applicable to all settings. Second, although randomized controlled trials provided prospectively defined study populations and systematic safety monitoring, many included trials had some concerns regarding risk of bias, mainly related to randomization reporting and outcome assessment procedures. Third, comparisons between vaccine types, doses, and regions were based primarily on between-study subgroup analyses and may be affected by confounding factors, including differences in vaccine products, populations, schedules, and surveillance practices. In particular, the vaccine-type analysis did not directly compare MMRV with MMR plus separately administered monovalent varicella vaccine and could not isolate the contribution of the varicella component to the observed differences in adverse event incidence. Fourth, serious adverse events were summarized over the available follow-up periods, generally days 0–42 after vaccination, because event-specific risk windows were not consistently reported across the source trials. Therefore, these observed incidences should not be interpreted as estimates within biologically specific risk windows for individual serious adverse events. Finally, the search was restricted to studies published from 1 January 2000, onward. This pragmatic cutoff may have excluded relevant trials conducted or published during the 1990s, including early licensure-era trials, and therefore may have limited the completeness of the available safety evidence.

## 5. Conclusions

This systematic review and meta-analysis provide descriptive estimates of the incidence of individual adverse events observed following varicella-containing vaccination in healthy children under active safety monitoring. Fever was the most frequently observed common adverse event, and injection-site reactions were also reported. Rare serious adverse events, including febrile seizures, anaphylaxis, immune thrombocytopenic purpura, and encephalitis, were infrequently observed in the included trials. Higher pooled incidences of fever and injection-site reactions were observed in MMRV than in monovalent varicella vaccine study groups; however, these between-study differences should not be interpreted as causal effects of vaccine type or as a comparison between MMRV and MMR plus separately administered monovalent varicella vaccine. Continued safety surveillance remains important for monitoring rare serious adverse events.

## Figures and Tables

**Table 1:** Characteristics of randomized controlled trials included in the systematic review and meta-analysis.

Author	Year	Country/Region	Vaccine	Surveillance	WHO Region
Shinefield [11]	2002	United States	VARIVAX	Active	AMR
Sun [12]	2003	China	Domestic live attenuated varicella vaccine (Shanghai Institute of Biological Products)	Active	WPR
Lin [13]	2004	China	Freeze-dried live attenuated varicella vaccine (Shanghai Institute/Changchun Institute)	Active	WPR
Shinefield [14]	2005	United States	ProQuad	Active	AMR
Knuf [15]	2006	Germany, Austria	Priorix-Tetra	Active	EUR
Lieberman [16]	2006	United States, Canada	ProQuad	Active	AMR
Reisinger [17]	2006	United States	ProQuad	Active	AMR
Bernstein [18]	2007	United States, Sweden	ProQuad (refrigerated formulation)	Active	Cross-region
Lu [19]	2007	China	Freeze-dried live attenuated varicella vaccine (Changsheng/Shanghai Institute)	Active	WPR
Nolan [20]	2008	Australia/United States	Varivax	Active	Cross-region
Schuster [21]	2008	Germany	Priorix-Tetra	Active	EUR
Ferrera [22]	2009	France, Italy	VARIVAX refrigerated formulation (Oka/Merck; Sanofi Pasteur MSD)	Active	EUR
Gillet [23]	2009	France, Italy, Germany	Priorix-Tetra	Active	EUR
Halperin [24]	2009	Canada, Italy	Priorix-Tetra	Active	Cross-region
Ma [25]	2009	China	Freeze-dried live attenuated varicella vaccine (Changchun Qijian)	Mixed	WPR
Cao [26]	2010	China	Freeze-dried live attenuated varicella vaccine (Changchun Qijian)	Active	WPR
Knuf [27]	2010	Germany	Priorix-Tetra	Active	EUR
Bai [28]	2011	China	Gelatin-free freeze-dried live attenuated varicella vaccine (Changchun BCHT)	Active	WPR
Watanaveeradej [29]	2011	Thailand	Varilrix (GSK), first-dose control for a dengue-vaccine trial	Active	SEAR
Xiao [30]	2011	China	Freeze-dried live attenuated varicella vaccine (Changchun Qijian)	Mixed	WPR
Tang [31]	2012	China	Gelatin-free freeze-dried live attenuated varicella vaccine (Changchun BCHT)	Active	WPR
Zhang [32]	2012	China	Freeze-dried live attenuated varicella vaccine (Changchun Qijian)	Mixed	WPR
Huang [33]	2013	Taiwan, China; Singapore	GSK MMRV (new measles/rubella working seeds; Priorix-Tetra type)	Active	WPR
Prymula [34]	2014	Czech Republic, Greece, Italy, Lithuania, Norway, Poland, Romania, Russia, Slovakia, Sweden	Priorix-Tetra	Active	EUR
Lalwani [35]	2015	India	Vammrix (Priorix-Tetra)	Active	SEAR
Mitra [36]	2015	India	VR 795 Oka strain	Active	SEAR
Zhu [37]	2015	India	Freeze-dried live attenuated varicella vaccine (Changchun BCHT)	Active	SEAR
Durando [38]	2016	Italy	Priorix-Tetra	Active	EUR
Prymula [39]	2016	Czech Republic, Hungary	Varilrix (HSA-free)	Active	EUR
Chen [40]	2017	China	Live attenuated varicella vaccine (GlaxoSmithKline)	Active	WPR
Chu [41]	2018	China	Freeze-dried live attenuated varicella vaccine (Shanghai Institute of Biological Products)	Active	WPR
Senders [42]	2018	United States	VARIVAX NSP	Active	AMR
Faust [43]	2019	Multinational	Varilrix (HSA-free)	Active	Cross-region
Haas [44]	2019	France	ProQuad	Active	EUR
Hao [45]	2019	China	Sinovac (Dalian) live attenuated varicella vaccine, human diploid SV-1 cells	Active	WPR
Huang [46]	2019	China	Monovalent varicella vaccine	Active	WPR
Li [47]	2019	China	Live attenuated varicella vaccine	Active	WPR
Reisinger [48]	2019	United States	VARIVAX	Active	AMR
Choi [49]	2021	South Korea, Thailand	MG1111/BARYCELA	Active	Cross-region
Choi [50]	2023	South Korea/Philippines/Mexico	NBP608/SKYVaricella (SK Bioscience)	Active	Cross-region
Sun [51]	2023	China	Sinovac (Dalian) live attenuated varicella vaccine	Active	WPR
Nazaire-Bermal [52]	2025	Philippines	Live attenuated varicella vaccine (Sinovac Dalian)	Active	WPR
Wang [53]	2025	China	Beijing Minhai freeze-dried live attenuated varicella vaccine	Active	WPR

**Table 2:** Random-effects GLMM pooled incidence of adverse events.

Outcome	Study Arms	Events/Participants	Pooled Incidence *, %	95% CI, %	I^2^, %	tau^2^	95% Prediction Interval, %
Fever	45	5261/31,472	11.580	6.786–19.068	98.8	3.9000	0.233–88.003
Redness/erythema	36	2192/23,792	2.742	1.100–6.667	96.2	7.0756	0.012–87.174
Cough	10	295/6754	2.292	0.488–10.079	98.8	5.7143	0.008–87.526
Pain	39	2215/26,057	2.191	0.932–5.061	97.0	6.6691	0.011–81.836
Swelling	40	901/27,299	0.815	0.333–1.982	90.9	6.5974	0.004–61.696
Generalized rash	36	612/22,587	0.407	0.161–1.021	97.1	5.9381	0.003–38.663
Gastrointestinal symptoms	36	840/26,619	0.058	0.011–0.305	94.9	12.5484	0.000–48.595
Injection-site rash	29	27/19,299	0.048	0.012–0.190	0.0	4.5564	0.000–4.556
Irritability	27	686/15,278	0.039	0.003–0.471	91.4	19.9466	0.000–84.601
Fatigue	29	304/22,688	0.010	0.001–0.137	94.9	14.0724	0.000–26.314

* Pooled incidence is the inverse logit of the mean study-specific logit from the random-effects GLMM.

**Table 3:** GRADE certainty of evidence for key safety questions.

Outcome/Question	Evidence	Effect Estimate	Certainty	Downgrading Rationale
Fever, MMRV	Supplementary Materials Section S9.3; 14 groups, 8994 participants	43.44% (95% CI 32.07–55.53)	Moderate	Inconsistency −1 (residual I^2^ = 98.5%; τ^2^ = 0.85 vs. overall 3.90). Between-study comparison
Local reactions, MMRV	Between-study subgroup (Results 3.4; Supplementary Materials Section S9.3)	Pain 12.27%; redness 16.50%; swelling 7.02%	Moderate	Inconsistency −1. Between-study comparison
Fever, overall (mixed products)	45 arms; 5261/31,472	11.58% (95% CI 6.79–19.07)	Low	Inconsistency −2 (I^2^ = 98.8%; 95% PI 0.23–88.00)
Fever, monovalent varicella vaccine	Supplementary Materials Section S9.3; 28 groups, 21,551 participants	5.29% (95% CI 2.62–10.40)	Moderate	Inconsistency −1 (residual I^2^ = 96.0%). Between-study comparison
Local reactions, overall (pain/redness/swelling)	39/36/40 arms; 2215/26,057; 2192/23,792; 901/27,299	2.19%/2.74%/0.82%	Low	Inconsistency −2 (I^2^ = 97.0%, 96.2%, 90.9%; wide PIs)
Local reactions, monovalent	Between-study subgroup (Results 3.4; Supplementary Materials Section S9.3)	Pain 0.91%; redness 0.69%; swelling 0.30%	Moderate	Inconsistency −1. Between-study comparison
Febrile seizure, overall	31 arms; 21/21,241	0.0989% (exact 95% CI 0.0612–0.1511)	Moderate	Imprecision −1 for the exact rate; CI entirely <0.5%
Encephalitis, overall	24 arms; 0/17,128	0 (exact upper 95% CI 0.0215%)	High	None for the <0.1% threshold. Zero events ≠ zero risk

Overall pooled estimates and vaccine-type-stratified estimates were assessed separately.

## Data Availability

The original contributions presented in this study are included in the article/Supplementary Materials. Further inquiries can be directed to the corresponding author.

## Supplementary Materials

The following supporting information can be downloaded at: https://www.mdpi.com/article/10.3390/vaccines14100910/s1, Supplementary Materials Section S1: Detailed Search Strategies; Supplementary Materials Section S2: Detailed Eligibility Criteria; Supplementary Materials Section S3: Outcome Definitions and Observation Windows; Supplementary Materials Section S4: Data Extraction and Data Handling Rules; Supplementary Materials Section S5: Detailed Statistical Analysis; Supplementary Materials Section S6: Risk of Bias and Certainty of Evidence; Supplementary Materials Section S7: Additional Data Handling and Analytical Considerations; Supplementary Materials Section S8: Preferred Reporting Items for Systematic Reviews and Meta-Analyses (PRISMA) 2020 Checklist; Supplementary Materials Section S9: Supplementary Results.
