# Supplementary material for: Adverse Events Following Varicella-Containing Vaccination in Healthy Children: A Systematic Review and Meta-Analysis
Source: Vaccines (Basel). 2026 Oct 20;14(10):910. doi: 10.3390/vaccines14100910 (PMC13709509; doi:10.3390/vaccines14100910)
Supplement: Supplementary file 1 [file vaccines-14-00910-s001.zip › vaccines-4607475-supplementary.pdf]

Supplementary Materials

Section S1. Detailed Search Strategies 3
S1.1 Information sources 3
S1.2 Search concepts 3
S1.3 Database-specific search strategies 4
Section S2. Detailed Eligibility Criteria 6
S2.1 Population 6
S2.2 Vaccines 6
S2.3 Study design 6
S2.4 Safety monitoring 7
S2.5 Outcome reporting 7
S2.6 Concomitant vaccination 8
S2.7 Publication and report eligibility 8
Section S3. Outcome Definitions and Observation Windows 9
S3.1 Outcomes of interest 9
S3.2 Observation windows 10
S3.3 Outcome definitions 10
S3.4 Overall adverse-event rate 11
Section S4. Data Extraction and Data Handling Rules 11
S4.1 Data extraction 11
S4.2 Study groups and multiple-arm trials 12
S4.3 Vaccination dose 12
S4.4 Safety analysis denominator 12
S4.5 Missing and unclear values 12
S4.6 Percentages and reconstructed event counts 12
S4.7 Rare events 13
Section S5. Detailed Statistical Analysis 13
S5.1 Software 13
S5.2 Meta-analysis of common adverse events 13
S5.3 Statistical heterogeneity 14
S5.4 Vaccine-type subgroup analysis 14
S5.5 Dose subgroup analysis 14
S5.6 WHO-region exploratory analysis 15
S5.7 Rare serious adverse events 15
S5.8 Sensitivity analysis 16
S5.9 Small-study effects 16
S5.10 Statistical significance 16
Section S6. Risk of Bias and Certainty of Evidence 17
S6.1 Risk of bias 17
S6.2 Considerations specific to adverse-event outcomes 18
S6.3 Certainty of evidence 18
Section S7. Additional Data Handling and Analytical Considerations 19

S7.1 Avoidance of double counting 19
S7.2 Within-report aggregation 19
S7.3 Interpretation of between-study subgroup differences 19
S7.4 Data not used for quantitative synthesis 20
Section S8. Preferred Reporting Items for Systematic Reviews and Meta-Analyses (PRISMA) 2020 Checklist 20
Section S9. Supplementary Results 24
S9.1 Reporting and Calculation of Overall Adverse Event Rates in Included Randomized Controlled Trials 24
S9.2 Fever Incidence After Varicella-containing Vaccination 26
S9.3 Incidence of Adverse Events Following Monovalent Varicella and MMRV Vaccination 27
S9.4 Adverse Events by Vaccination Dose 29
S9.5 Exploratory Random-effects GLMM Subgroup Analyses by WHO Region 30
S9.6 Rare Serious Adverse Events and Exact Clopper-Pearson Confidence Intervals 31
S9.7. Risk-of-bias Summary Across the 43 Included Trials 32
S9.8 Sensitivity Analyses for Fever Incidence 33
S9.9 Exploratory assessment of small-study effects 34
S9.9a Funnel plot of fever incidence. Study-group logits versus standard error; the vertical line is the random-effects GLMM pooled estimate 34
S9.9b Egger tests of funnel-plot asymmetry for common adverse events 34

Section S1. Detailed Search Strategies

S1.1 Information sources

Eight electronic databases were searched from January 1, 2000, to July 17, 2026: PubMed, Embase, CENTRAL, Web of Science, Scopus, CNKI, Wanfang Data, and VIP. The January 1, 2000 start was prespecified. Publications before that date were not searched. Earlier randomized trials, including licensure-era reports from the 1990s, are therefore outside this review.

The search strategy was developed to identify randomized controlled trials evaluating the safety of varicella-containing vaccines in children. Searches combined controlled vocabulary, where available, with free-text terms related to varicella vaccination and adverse events.

The English-language databases were searched first, followed by the Chinese databases. Search strategies were adapted to the indexing system and syntax of each database.

S1.2 Search concepts

The search strategy included terms representing two main concepts:

1. **Varicella-containing vaccination**, including monovalent varicella vaccines and measles–mumps–rubella–varicella (MMRV) vaccines; and
2. **Adverse events and vaccine safety**, including adverse events, adverse reactions, safety, local reactions, and systemic reactions.

Key English search terms included:

- “varicella vaccine”
- “varicella vaccin*”
- “chickenpox vaccine”
- “MMRV”
- “adverse event*”
- “adverse reaction*”
- “safety”
- “local reaction*”
- “systemic reaction*”

Equivalent Chinese terms were used for searches in CNKI, Wanfang Data, and VIP Database.

No date restriction was applied beyond the prespecified search period. The complete database-specific search strategies, including controlled vocabulary, free-text terms, field restrictions, truncation, and Boolean operators, are provided below.

S1.3 Database-specific search strategies

PubMed

("Chickenpox Vaccine"[Mesh] OR varicella vaccin*[tiab] OR chickenpox vaccin*[tiab] OR MMRV[tiab] OR "measles mumps rubella varicella"[tiab]) AND ("Drug-Related Side Effects and Adverse Reactions"[Mesh] OR adverse event*[tiab] OR adverse reaction*[tiab] OR side effect*[tiab] OR safety[tiab] OR post-vaccination reaction*[tiab] OR "adverse event following immunization"[tiab] OR AEFI[tiab] OR serious adverse event*[tiab] OR SAE[tiab] OR local reaction*[tiab] OR systemic reaction*[tiab])

Embase

('varicella vaccine'/exp/mj OR 'measles mumps rubella varicella vaccine'/exp/mj OR (((varicella:ti,ab AND vaccin*:ti,ab OR chickenpox:ti,ab) AND vaccin*:ti,ab OR mmrv:ti,ab OR 'measles mumps rubella varicella':ti,ab) AND vaccin*:ti,ab)) AND ('adverse drug reaction'/exp/mj OR 'vaccine safety'/exp/mj OR (((adverse:ti,ab AND event*:ti,ab OR adverse:ti,ab) AND reaction*:ti,ab OR side:ti,ab) AND effect*:ti,ab) OR safety:ti,ab OR 'post-vaccination reaction*':ti,ab OR 'adverse event following immunization':ti,ab OR aefi:ti,ab OR 'serious adverse event*':ti,ab OR sae:ti,ab OR 'local reaction*':ti,ab OR 'systemic reaction*':ti,ab)

Cochrane Library

#1 [mh "Chickenpox Vaccine"] 307

#2 [mh "Measles-Mumps-Rubella-Varicella Vaccine"] 0

#3 (varicella NEXT vaccin* OR chickenpox NEXT vaccin* OR MMRV OR "measles mumps rubella varicella" NEXT vaccin*):ti,ab,kw 346

#4 #1 OR #2 OR #3 481

#5 [mh "Drug-Related Side Effects and Adverse Reactions"] 5252

#6 (adverse NEXT event* OR adverse NEXT reaction* OR side NEXT effect* OR "post NEXT vaccination NEXT reaction*"):ti,ab,kw 339695

#7 ("adverse event following immunization" OR AEFI OR "serious NEXT adverse NEXT event*" OR SAE OR "local NEXT reaction*" OR "systemic NEXT reaction*"):ti,ab,kw 27654

#8 #5 OR #6 OR #7 362027

#9 #4 AND #8 212

Web of Science

TS=(varicella vaccin* OR chickenpox vaccin* OR MMRV OR "measles mumps rubella varicella" vaccin*) AND TS=(adverse event* OR adverse reaction* OR side effect* OR safety OR post-vaccination reaction* OR "adverse event following immunization" OR AEFI OR serious adverse event* OR SAE OR severe reaction* OR local reaction* OR systemic reaction*)

Scopus

TITLE-ABS-KEY(varicella vaccin* OR chickenpox vaccin* OR MMRV OR "measles mumps rubella varicella" vaccin*) AND TITLE-ABS-KEY(adverse event* OR adverse reaction* OR side effect* OR safety OR post-vaccination reaction* OR "adverse event following immunization" OR AEFI OR serious adverse event* OR SAE OR severe reaction* OR local reaction* OR systemic reaction*)

CNKI

SU=(Varicella vaccine + Live attenuated varicella vaccine + Measles-mumps-rubella-varicella vaccine + Combined measles-mumps-rubella-varicella vaccine + MMRV vaccine) AND SU=(Adverse reaction + Adverse event + Side effect + Vaccination reaction + Safety + Adverse reaction to vaccination + Vaccine adverse reaction + Post-vaccination adverse reaction + AEFI + Serious adverse reaction + Serious adverse event + SAE + Local reaction + Systemic reaction + Abnormal reaction)

Wanfang Data

Subject:(Varicella vaccine OR Live attenuated varicella vaccine OR Measles-mumps-rubella-varicella vaccine OR Combined measles-mumps-rubella-varicella vaccine OR MMRV vaccine) AND Subject:(Adverse reaction OR Adverse event OR Side effect OR Vaccination reaction OR Safety OR Adverse reaction to vaccination OR Vaccine adverse reaction OR Post-vaccination adverse reaction OR Serious adverse reaction OR Serious adverse event OR SAE OR Local reaction OR Systemic reaction OR Abnormal reaction)

VIP Database

M=(Varicella vaccine OR Live attenuated varicella vaccine OR Measles-mumps-rubella-varicella vaccine OR Combined measles-mumps-rubella-varicella vaccine OR MMRV vaccine) AND M=(Adverse reaction OR Adverse event OR Side effect OR Vaccination reaction OR Safety OR Adverse reaction to vaccination OR Vaccine adverse reaction OR Post-vaccination adverse reaction OR AEFI OR Serious adverse reaction OR Serious adverse event OR SAE OR Local reaction OR Systemic reaction OR Abnormal reaction)

Section S2. Detailed Eligibility Criteria

S2.1 Population

Eligible studies enrolled healthy children who were aged ≥12 months and <12 years at the time of vaccination.

Studies enrolling broader age ranges were eligible only when data for children aged ≥12 months and <12 years could be separately identified and extracted.

Studies involving children with underlying medical conditions, immunocompromising conditions, or other special populations were excluded when safety data for otherwise healthy children could not be separated.

One eligible trial (Knuf, 2010) reported a small proportion of children aged 11 months whose data could not be separated from those of children meeting the predefined age criterion. This study was retained because the deviation from the predefined lower age boundary was minimal. The potential influence of this inclusion was evaluated in a prespecified sensitivity analysis excluding the study.

S2.2 Vaccines

Eligible interventions were licensed vaccines containing a varicella component, including:

1. licensed monovalent live attenuated varicella vaccines; and
2. licensed measles–mumps–rubella–varicella (MMRV) vaccines.

Studies evaluating investigational, unlicensed, or non-target vaccines were excluded.

When multiple vaccine products or formulations were evaluated within the same trial, eligible vaccine groups were extracted separately.

S2.3 Study design

Eligible studies were randomized controlled trials, including individual-level and cluster randomized controlled trials.

Non-randomized studies, observational studies, case-control studies, cohort studies, cross-sectional studies, case series, case reports, and other non-randomized designs were excluded.

The restriction to randomized controlled trials was prespecified because the objective was to characterize reported adverse events under prospectively defined and actively monitored conditions. However, inclusion of an RCT did not imply that single-arm incidence estimates had causal interpretability when randomized comparator data were not incorporated.

S2.4 Safety monitoring

Eligible studies were required to use prospectively defined active or predominantly active safety monitoring.

Active monitoring methods included, but were not limited to:

- participant or caregiver-completed diary cards;
- prospectively recorded symptom diaries;
- caregiver-recorded adverse-event reports;
- scheduled post-vaccination visits;
- active telephone follow-up; and
- other prespecified follow-up procedures in which participants were actively contacted or assessed for adverse events.

Studies relying exclusively on spontaneous or passive reporting were excluded.

For studies using multiple safety-monitoring approaches, the overall monitoring strategy was considered, and studies were eligible when safety ascertainment was predominantly active and prospectively defined.

S2.5 Outcome reporting

A study was eligible for quantitative extraction if it reported at least one prespecified adverse event and provided:

- the number of participants experiencing the event; and
- the corresponding safety analysis denominator

for the same study group, vaccination dose, and prespecified observation period.

Reports providing only qualitative statements, such as "no safety concerns were observed," without verifiable event counts and denominators were excluded from quantitative analysis.

Studies reporting percentages were considered eligible when the corresponding safety denominator allowed the event count to be verified or reasonably reconstructed. Estimates derived from rounded percentages were identified as estimated values and were not used when a reliable numerator could not be established.

S2.6 Concomitant vaccination

Studies were excluded when event data could not be assigned to the relevant vaccine or vaccination-strategy group, or when the corresponding safety denominator was unavailable. When concomitant vaccines were administered and events were reported for the combined strategy, the data were interpreted as events following that strategy and not as varicella-vaccine-specific adverse reactions.

When concomitant vaccines were administered but adverse events were separately reported for the eligible varicella-containing vaccine group, the data were considered eligible.

S2.7 Publication and report eligibility

Multiple publications originating from the same trial were treated as reports of a common trial. Duplicate reports were identified and linked during study selection and data extraction to avoid double counting of participants.

Reviews, systematic reviews, meta-analyses, protocols, editorials, commentaries, letters without sufficient original data, and conference abstracts without sufficient information for quantitative extraction were excluded.

Section S3. Outcome Definitions and Observation Windows

S3.1 Outcomes of interest

The prespecified outcomes were individual adverse events following immunization. Extracted event counts were not classified as vaccine-attributable adverse reactions. Where a source trial reported its own causality assessment, that assessment was retained as study-level information and was not used to reclassify events in the pooled analyses.

Common injection-site adverse events

- Injection-site pain
- Injection-site erythema
- Injection-site swelling
- Injection-site rash

Common systemic adverse events

- Fever
- Generalized rash
- Gastrointestinal symptoms
- Fatigue
- Irritability
- Cough

Rare serious adverse events

- Febrile seizures
- Anaphylaxis
- Immune thrombocytopenic purpura (ITP)
- Encephalitis

S3.2 Observation windows

The following descriptive observation windows were prespecified before data extraction:

Outcome category	Prespecified observation window
Injection-site adverse events	0–7 days after vaccination
Fever and common systemic adverse events	0–14 days after vaccination
Serious adverse events	0–42 days after vaccination, used jointly for febrile seizure, anaphylaxis, ITP, and encephalitis because separate biological windows were not reported

The day of vaccination was considered day 0. Injection-site events were sought over days 0–7, and fever and other common systemic events over days 0–14. Febrile seizure, anaphylaxis, immune thrombocytopenic purpura, and encephalitis were sought over one shared window, days 0–42. These four events have different biologically plausible risk windows, but the source trials generally did not publish counts inside separate windows. The shared window records the follow-up that could be extracted. It is not a claim that one biological window fits all four events.

When a study reported multiple observation windows for the same outcome, the observation period that most closely matched the prespecified window was selected.

When more than one eligible observation period was available, data were preferentially selected according to the following hierarchy:

1. the observation window exactly matching the prespecified window;
2. the observation window that most closely approximated the prespecified window; and
3. a longer observation window only when the event ascertainment could be clearly attributed to the prespecified period.

When the reported observation period did not allow reliable alignment with the prespecified window, the data were not included in the corresponding quantitative synthesis.

S3.3 Outcome definitions

The original study definitions were retained during data extraction. Where terminology varied across studies, clinically equivalent terms were mapped to the prespecified outcome categories when the definition and observation period were sufficiently comparable.

Differences in outcome definitions and ascertainment procedures were recorded and considered when assessing statistical heterogeneity and certainty of evidence.

S3.4 Overall adverse-event rate

An overall adverse-event rate was not derived by summing the numbers of participants experiencing individual adverse events because a participant could experience more than one adverse event.

Many included studies did not report the proportion of participants experiencing at least one adverse event. Therefore, an overall adverse-event rate could not be consistently calculated across studies and was not quantitatively synthesized.

When an overall adverse-event rate was directly reported in an original study, it was not reconstructed from individual symptom counts.

Section S4. Data Extraction and Data Handling Rules

S4.1 Data extraction

Two reviewers independently extracted data using a standardized data-extraction form after completion of title/abstract and full-text screening. Trial reports from the same study were linked to avoid double counting. Disagreements were resolved by discussion or consultation with a third reviewer. Before analysis, extracted records were checked for consistency of trial identifiers, vaccine groups, dose, observation period, event counts, and safety denominators.

The following information was extracted during the standardized data-management process:

- first author;
- publication year;
- trial identifier, when available;
- study country or countries;
- trial implementation site;
- WHO region;
- study design;
- sample size;
- vaccine type;
- vaccine product, when reported;
- vaccination dose;
- concomitant vaccination;
- safety-monitoring method;
- observation period;
- adverse-event definition;
- number of participants experiencing each adverse event;
- safety analysis denominator;
- information relevant to risk-of-bias assessment; and whether a usable comparator group was available for the reported outcome.

S4.2 Study groups and multiple-arm trials

For multi-arm randomized trials, eligible vaccine groups were extracted separately when they represented independent study groups.

Study groups were not automatically combined across vaccine types or vaccination doses.

When multiple eligible groups from the same report were mutually independent and had comparable outcome definitions and observation periods, within-study aggregation was permitted for descriptive purposes when appropriate.

S4.3 Vaccination dose

Adverse-event data were extracted separately according to vaccination dose whenever dose-specific data were available.

First-dose and second-dose data from the same participants were not combined because the observations were not statistically independent.

S4.4 Safety analysis denominator

The denominator used for each outcome was the safety analysis population corresponding to the same study group, vaccination dose, and observation period.

The safety analysis denominator was preferred over the total randomized population when the study provided a specific safety population.

S4.5 Missing and unclear values

Missing values, not-reported values, and unclear coding were not interpreted as zero events.

A value was coded as zero only when the original report explicitly indicated that no events occurred or reported a zero event count.

Codes such as “999,” “NR,” or other study-specific missing-value codes were treated as missing unless the original study explicitly defined them as zero.

S4.6 Percentages and reconstructed event counts

When both an event count and denominator were reported, the event count was used directly.

When only a percentage and the corresponding denominator were reported, the event count was reconstructed only when the resulting value could be verified against the reported percentage and did not introduce material ambiguity.

When rounding prevented reliable reconstruction of the numerator, the study was not included in the corresponding quantitative analysis.

S4.7 Rare events

For rare serious adverse events, event counts were extracted directly from the original reports whenever available.

Zero events were retained as zero-event observations and were not converted to missing values.

Section S5. Detailed Statistical Analysis

S5.1 Software

All statistical analyses were conducted using R version 4.6.1 (R Foundation for Statistical Computing, Vienna, Austria) with the **meta** package.

The statistical code used for the primary analyses and prespecified sensitivity analyses was retained by the study team.

S5.2 Meta-analysis of common adverse events

Each prespecified common adverse event was analyzed separately.

Event counts and corresponding safety denominators were used to estimate incidence proportions.

Random-effects meta-analysis of single proportions was performed using a generalized linear mixed model with a logit link and a random intercept. The model-based estimate summarized the distribution of study-specific proportions and was not equivalent to a participant-weighted crude risk or a vaccine-attributable risk. Crude event counts and denominators were also reported for verification.

The pooled estimate was back-transformed to the proportion scale and reported with a two-sided 95% confidence interval. Back-transformation of the mean study-specific logit does not produce the crude proportion (total events divided by total participants). With large τ^2, the pooled incidence can be far below the crude proportion, as for gastrointestinal symptoms (840/26,619; crude proportion 3.16%; pooled incidence 0.058%) and irritability (686/15,278; crude proportion 4.49%; pooled incidence 0.039%). Where the two summaries diverge, the pooled incidence is not read as the average risk.

The analysis unit was the study group rather than the individual participant.

S5.3 Statistical heterogeneity

Between-study heterogeneity was assessed using:

- I^2, representing the proportion of total variability attributable to between-study heterogeneity; and
- τ^2, representing the estimated between-study variance on the model scale.

When sufficient studies were available, 95% prediction intervals were calculated to describe the expected range of true incidence proportions across comparable study settings.

Because single-proportion meta-analyses may exhibit substantial heterogeneity arising from differences in populations, vaccine products, outcome definitions, monitoring procedures, and observation periods, heterogeneity statistics were interpreted together with the clinical and methodological characteristics of the included studies.

S5.4 Vaccine-type subgroup analysis

A prespecified subgroup analysis was conducted according to vaccine type:

1. monovalent varicella vaccine; and
2. MMRV vaccine.

Subgroup-specific incidence proportions and 95% CIs were estimated using the same random-effects GLMM framework.

Differences between subgroups were assessed using the test for subgroup differences under the random-effects model.

Because vaccine type may be associated with other study-level characteristics, subgroup estimates were interpreted as indirect descriptive comparisons rather than direct causal comparisons. The clinically relevant comparison of MMRV versus separately administered MMR plus monovalent varicella vaccine was not reconstructed from the available extraction. MMRV contains three additional live antigens, so a higher single-arm summary does not identify an effect of the varicella component.

S5.5 Dose subgroup analysis

Prespecified subgroup analyses were conducted according to vaccination dose:

- first dose;
- second dose.

First- and second-dose observations from the same participants were not combined.

When the same study contributed multiple dose-specific groups, each eligible dose-specific group was analyzed separately.

S5.6 WHO-region exploratory analysis

An exploratory subgroup analysis was conducted according to WHO region:

- African Region (AFR);
- Region of the Americas (AMR);
- South-East Asia Region (SEAR);
- European Region (EUR);
- Eastern Mediterranean Region (EMR); and
- Western Pacific Region (WPR).

WHO region was assigned according to the location where the trial was implemented rather than the institutional affiliation of the first or corresponding author.

When a study was conducted in two or more WHO regions, it was classified as a multi-region study.

The WHO-region analysis was restricted to common adverse events for which sufficient data were available.

For regions represented by at least two independent study groups, random-effects GLMMs were used to estimate pooled incidence proportions.

When only one study group was available within a region, the observed incidence proportion was reported together with an exact 95% CI calculated using the Clopper–Pearson method, without random-effects pooling.

Regions without eligible study groups were considered to have insufficient evidence.

Differences between WHO-region subgroups were assessed using the test for subgroup differences under the random-effects model when applicable.

Because WHO region and vaccine type may be correlated across studies, WHO-region findings were interpreted as exploratory inter-study comparisons and were not considered evidence of regional differences in vaccine safety or causal effects.

Rare serious adverse events were not subjected to WHO-region subgroup analyses because of their low frequency and sparse data.

S5.7 Rare serious adverse events

Rare serious adverse events, including febrile seizures, anaphylaxis, ITP, and encephalitis, were summarized using events reported during available study-specific follow-up and the corresponding safety population. Exact two-sided 95% CIs were calculated using the Clopper–Pearson method.

These proportions were descriptive and were not interpreted as outcome-specific biological or vaccine-attributable risks. All four events were summarized on the shared 0–42 day window in Section 3.2, because the source trials generally did not report separate counts inside event-specific biological windows. That shared window is not a claim that the four events have the same biological risk period.

S5.8 Sensitivity analysis

Two prespecified sensitivity analyses were conducted.

First, the study by Knuf et al.[27], which included a small proportion of children aged 11 months who could not be separated from the eligible population, was excluded to assess the influence of the age-boundary deviation.

Second, a sensitivity analysis for fever excluded studies judged to have an overall high trial-level risk of bias according to RoB 2. Because the exclusion was not fully outcome-specific, this analysis was considered exploratory.

The resulting pooled estimates were compared with the primary analyses to assess the robustness of the findings.

S5.9 Small-study effects

When at least 10 study groups were available for an outcome, funnel plots were examined and Egger's linear regression test was used to explore potential small-study effects.

These analyses were considered exploratory. Asymmetry in funnel plots or a statistically significant Egger test was not interpreted as direct evidence of publication bias because single-proportion meta-analysis may be affected by heterogeneity, differences in study size, outcome prevalence, and the presence of rare or zero-event outcomes.

S5.10 Statistical significance

All statistical tests were two-sided, and $P<0.05$ was considered statistically significant.

Section S6. Risk of Bias and Certainty of Evidence

S6.1 Risk of bias

Risk of bias was assessed independently by two reviewers using the Cochrane RoB 2 tool for randomized controlled trials.

The following five domains were assessed:

1. bias arising from the randomization process;
2. bias due to deviations from intended interventions;
3. bias due to missing outcome data;
4. bias in measurement of the outcome; and
5. bias in selection of the reported result.

Each domain was judged as:

- low risk of bias;
- some concerns; or
- high risk of bias.

An overall trial-level risk-of-bias judgment was assigned according to the RoB 2 algorithm. The scored profile is anchored to solicited common adverse events and is an overview of that evidence. It is not an outcome-specific rating for febrile seizure, anaphylaxis, ITP, or encephalitis, and those rare events were not given a second numerical profile. Randomization does not depend on the outcome. Missing data and outcome measurement can differ, and the rare-event results remain limited by sparse counts and by the shared 0–42 day window.

Disagreements were resolved by discussion or consultation with a third reviewer.

S6.2 Considerations specific to adverse-event outcomes

Because the review focused on actively monitored safety outcomes, particular attention was paid to the completeness and consistency of outcome ascertainment.

The solicited-outcome profile does not by itself rate every safety outcome. Features that can differ across outcomes are whether:

- the outcome measurement method was prospectively defined;
- the same or comparable ascertainment procedure was applied across study groups;
- the observation period was clearly specified;
- missing outcome data were likely to affect the estimated incidence; and
- the reported analysis appeared to be selectively chosen from among multiple eligible analyses.

Differences in outcome ascertainment or definitions were considered in conjunction with between-study heterogeneity when evaluating the certainty of evidence.

S6.3 Certainty of evidence

The certainty of evidence for each outcome was assessed using the GRADE approach.

Because all included studies were randomized controlled trials, evidence initially started at high certainty.

Evidence was downgraded, when appropriate, for:

- risk of bias;
- inconsistency;
- indirectness;
- imprecision; and
- publication bias.

The assessment was conducted for the relevant descriptive estimate. Because the principal synthesis used single-arm data, certainty judgments were interpreted as describing the reliability of reported incidence evidence rather than certainty of a causal vaccine safety effect. They do not rate a causal effect of vaccination compared with no vaccination, placebo, or an alternative schedule.

Differences in outcome definitions, outcome ascertainment, and observation windows were considered primarily under inconsistency when they contributed to heterogeneity across studies. The same limitation was not automatically counted again as indirectness.

Risk-of-bias judgments were considered at the outcome level rather than by mechanically applying the overall distribution of RoB 2 judgments across all included studies.

Evidence was not downgraded solely because a substantial proportion of studies were judged to have some concerns when the concerns were unlikely to materially affect the specific outcome estimate.

Imprecision was assessed based on the precision of the effect estimate and the width of the confidence interval, taking into account the clinical interpretation of the estimated incidence rather than applying an incidence threshold alone.

Evidence was not downgraded for publication bias solely on the basis of Egger's test or funnel-plot asymmetry.

Section S7. Additional Data Handling and Analytical Considerations

S7.1 Avoidance of double counting

Individual adverse-event categories were analyzed separately. The numbers of participants experiencing different adverse events were not added together because a single participant could experience multiple adverse events. Participants were considered separately across vaccination doses only when dose-specific observations were explicitly reported. First- and second-dose observations from the same participants were not combined into a single incidence estimate.

S7.2 Within-report aggregation

When multiple study groups from the same report were considered for descriptive aggregation, groups were combined only when:

1. the groups were mutually independent;
2. the outcome definition was comparable;
3. the observation window was comparable;
4. the vaccination dose was comparable; and
5. the safety denominators corresponded to the same outcome ascertainment framework.

Groups that did not meet these conditions were retained separately.

S7.3 Interpretation of between-study subgroup differences

Subgroup analyses by vaccine type and WHO region were interpreted as comparisons among study groups rather than direct comparisons among individual participants.

In particular, higher or lower incidence estimates between vaccine-type subgroups were not interpreted as evidence that one vaccine type caused more or fewer adverse events than another unless supported by an appropriate within-trial randomized comparison.

S7.4 Data not used for quantitative synthesis

Data were not included in quantitative synthesis when:

- the numerator could not be verified;
- the corresponding safety denominator was unavailable;
- the outcome definition was incompatible with the prespecified outcome;
- the observation window could not be aligned with the prespecified window;
- events following varicella-containing vaccination could not be distinguished from events following concomitant vaccination;
- the same participants would have been counted more than once in the same analysis.

Such information was retained for study-level assessment where appropriate but was not used to generate pooled incidence estimates.

Section S8. Preferred Reporting Items for Systematic Reviews and Meta-Analyses (PRISMA) 2020 Checklist

Source: Page MJ et al. PRISMA 2020 statement (BMJ 2021;372:n71).

Section and Topic	Item #	Checklist item	Location where item is reported
**TITLE**			
Title	1	Identify the report as a systematic review.	Title page
**ABSTRACT**			
Abstract	2	See the PRISMA 2020 for Abstracts checklist.	Abstract: Background, Methods, Results and Conclusions
**INTRODUCTION**			
Rationale	3	Describe the rationale for the review in the context of existing knowledge.	Introduction, paragraphs 3–4: limitations of existing evidence and previous reviews
Objectives	4	Provide an explicit statement of the objective(s) or question(s) the review addresses.	Introduction, final paragraph: review objectives
**METHODS**			
Eligibility criteria	5	Specify the inclusion and exclusion criteria for the review and how studies were grouped for the syntheses.	Methods 2.3; Supplementary Section 2.1–2.7
Information sources	6	Specify all databases, registers, websites, organisations, reference lists and other sources searched or consulted to identify studies. Specify the date when each source was last searched or consulted.	Methods 2.2; Supplementary Section 1.1
Search	7	Present the full search strategies for all databases, registers	Methods 2.2; Supplementary Section 1.3

Section and Topic	Item #	Checklist item	Location where item is reported
strategy		and websites, including any filters and limits used.	
Selection process	8	Specify the methods used to decide whether a study met the inclusion criteria of the review, including how many reviewers screened each record and each report retrieved, whether they worked independently, and if applicable, details of automation tools used in the process.	Methods 2.4: two reviewers independently screened titles, abstracts and full texts; disagreements resolved by a third reviewer
Data collection process	9	Specify the methods used to collect data from reports, including how many reviewers collected data from each report, whether they worked independently, any processes for obtaining or confirming data from study investigators, and if applicable, details of automation tools used in the process.	Methods 2.4: two reviewers independently extracted data using a standardized form
Data items	10a	List and define all outcomes for which data were sought. Specify whether all results that were compatible with each outcome domain in each study were sought (e.g. for all measures, time points, analyses), and if not, the methods used to decide which results to collect.	Methods 2.5; Supplementary Section 3.1–3.3
	10b	List and define all other variables for which data were sought (e.g. participant and intervention characteristics, funding sources). Describe any assumptions made about any missing or unclear information.	Methods 2.4; Supplementary Sections 2, 3 and 7
Study risk of bias assessment	11	Specify the methods used to assess risk of bias in the included studies, including details of the tool(s) used, how many reviewers assessed each study and whether they worked independently, and if applicable, details of automation tools used in the process.	Methods 2.6: Cochrane RoB 2; two independent reviewers
Effect measures	12	Specify for each outcome the effect measure(s) (e.g. risk ratio, mean difference) used in the synthesis or presentation of results.	Methods 2.7: incidence proportions and 95% CIs; exact CIs for rare events
Synthesis methods	13a	Describe the processes used to decide which studies were eligible for each synthesis (e.g. tabulating the study intervention characteristics and comparing against the planned groups for each synthesis (item #5)).	Methods 2.7; Supplementary Section 7.2
	13b	Describe any methods required to prepare the data for presentation or synthesis, such as handling of missing summary statistics, or data conversions.	Methods 2.4 and 2.7; Supplementary Sections 3 and 7
	13c	Describe any methods used to tabulate or visually display results of individual studies and syntheses.	Results 3.2–3.9; Tables 1–3; Figures 1 and 2; Supplementary Sections 9.1–9.9
	13d	Describe any methods used to synthesize results and provide a rationale for the choice(s). If meta-analysis was performed,	Methods 2.7: random-effects GLMM, logit link, I^2, τ^2, prediction intervals and R meta

Section and Topic	Item #	Checklist item	Location where item is reported
		describe the model(s), method(s) to identify the presence and extent of statistical heterogeneity, and software package(s) used.	package
	13e	Describe any methods used to explore possible causes of heterogeneity among study results (e.g. subgroup analysis, meta-regression).	Methods 2.7: subgroup analyses by vaccine type, vaccination dose and WHO region
	13f	Describe any sensitivity analyses conducted to assess robustness of the synthesized results.	Methods 2.7; Results 3.9; Supplementary Section 9.8
Reporting bias assessment	14	Describe any methods used to assess risk of bias due to missing results in a synthesis (arising from reporting biases).	Methods 2.7: funnel plots and Egger's test when at least 10 study groups were available
Certainty assessment	15	Describe any methods used to assess certainty (or confidence) in the body of evidence for an outcome.	Methods 2.6; Results 3.10; Table 3
**RESULTS**			
Study selection	16a	Describe the results of the search and selection process, from the number of records identified in the search to the number of studies included in the review, ideally using a flow diagram.	Results 3.1 and Figure 1: 7,486 records identified; 43 trials included
	16b	Cite studies that might appear to meet the inclusion criteria, but which were excluded, and explain why they were excluded.	Results 3.1 and Figure 1: 463 full-text reports excluded; reasons summarized
Study characteristics	17	Cite each included study and present its characteristics.	Results 3.2; Table 1
Risk of bias in studies	18	Present assessments of risk of bias for each included study.	Results 3.8; Figure 2; Supplementary Section 9.7
Results of individual studies	19	For all outcomes, present, for each study: (a) summary statistics for each group (where appropriate) and (b) an effect estimate and its precision (e.g. confidence/credible interval), ideally using structured tables or plots.	Results 3.3–3.7; Table 2; Supplementary Sections 9.1–9.6
Results of syntheses	20a	For each synthesis, briefly summarise the characteristics and risk of bias among contributing studies.	Results 3.2 and 3.8
	20b	Present results of all statistical syntheses conducted. If meta-analysis was done, present for each the summary estimate and its precision (e.g. confidence/credible interval) and measures of statistical heterogeneity. If comparing groups, describe the direction of the effect.	Results 3.3–3.7; Tables 2–3; Supplementary Sections 9.2–9.6
	20c	Present results of all investigations of possible causes of heterogeneity among study results.	Results 3.4–3.6
	20d	Present results of all sensitivity analyses conducted to assess the robustness of the synthesized results.	Results 3.9; Supplementary Section 9.8

Section and Topic	Item #	Checklist item	Location where item is reported
Reporting biases	21	Present assessments of risk of bias due to missing results (arising from reporting biases) for each synthesis assessed.	Results 3.9; Supplementary Section 9.9a,b
Certainty of evidence	22	Present assessments of certainty (or confidence) in the body of evidence for each outcome assessed.	Results 3.10; Table 3
**DISCUSSION**			
Discussion	23a	Provide a general interpretation of the results in the context of other evidence.	Discussion, paragraphs 1–6
	23b	Discuss any limitations of the evidence included in the review.	Discussion, limitations paragraph
	23c	Discuss any limitations of the review processes used.	Discussion, limitations paragraph; Supplementary Section 7
	23d	Discuss implications of the results for practice, policy, and future research.	Discussion, final paragraphs and Conclusion
**OTHER INFORMATION**			
Registration and protocol	24a	Provide registration information for the review, including register name and registration number, or state that the review was not registered.	Not registered; no registration number reported
	24b	Indicate where the review protocol can be accessed, or state that a protocol was not prepared.	Protocol developed prospectively; detailed methods provided in the Supplementary Section
	24c	Describe and explain any amendments to information provided at registration or in the protocol.	No protocol amendments reported; data-coding revisions documented in Supplementary Sections 7 and 9.4
Support	25	Describe sources of financial or non-financial support for the review, and the role of the funders or sponsors in the review.	Not reported in the manuscript or appendix
Competing interests	26	Declare any competing interests of review authors.	Not reported in the manuscript or appendix
Availability of data, code and other materials	27	Report which of the following are publicly available and where they can be found: template data collection forms; data extracted from included studies; data used for all analyses; analytic code; any other materials used in the review.	Data, code and other materials availability not reported; extraction procedures described in the Supplementary Section

Section S9. Supplementary Results

S9.1 Reporting and Calculation of Overall Adverse Event Rates in Included Randomized Controlled Trials

Study	Citation	Country/region	Groups combined	Any-AE definition	Observation window	Study-level rate	Numerator source
Lieberman 2006	[16]	United States, Canada	Three ProQuad lots + M-M-R II with VARIVAX	One or more adverse experiences	Days 0–42	81.4% (3,137/3,853)	Exact
Reisinger 2006	[17]	United States	ProQuad + M-M-R II with VARIVAX (varicella-containing groups)	One or more adverse experiences	Days 1–43	≈ 76.9%‡	Percentage only
Nolan 2008	[20]	Australia, United States	MMR + varicella, with or without LAIV	Any AE	42 days after dose 1	47.5% (382/804)	Exact
Ferrera 2009	[22]	France, Italy	VARIVAX; pooled crossover periods	At least one AE	42 days	71.9% (360/501)	Exact
Halperin 2009	[24]	Canada, Italy	MMRV + MMR with varicella	Any solicited or unsolicited AE	43 days	≈ 68.0%‡	Percentage only
Huang 2013	[33]	Taiwan, China; Singapore	MMRV new working seed + MMRV comparator	Overall solicited and unsolicited symptoms	43 days after each dose	Dose 1: ≈ 87.3%‡; Dose 2: ≈ 69.7%‡	Percentage only
Mitra 2015	[36]	India	VR 795 + control	Any local and/or	Six weeks	≈ 43.5%‡	Percenta

Study	Citation	Country/region	Groups combined	Any-AE definition	Observation window	Study-level rate	Numerator source
			varicella vaccines	systemic AE			ge only
Senders 2018	[42]	United States	VARIVAX NSP + standard VARIVAX	One or more AEs	Days 1–42 after each dose	Dose 1: 72.1% (421/584); Dose 2: 58.8% (325/553)	Exact
Reisinger 2019	[48]	United States	Three VARIVAX formulations with MMR	One or more AEs	Days 0–42	85.2% (792/930)	Exact
Nazaire-Bermal 2025	[52]	Philippines	Test + active-control varicella vaccines	Any AE	42 days	≈ 68.0%‡	Percentage only
Wang 2025	[53]	China	Test + active-control varicella vaccines	Any AE	Within 42 days	28.0% (336/1,198)	Exact

Notes: AE, adverse event; Any-AE, at least one adverse event experienced by a participant within the stated observation window; MMR, measles–mumps–rubella vaccine; MMRV, measles–mumps–rubella–varicella vaccine; LAIV, live attenuated influenza vaccine; NSP, non-standard process. Exact n/N values were transcribed or combined only when independent varicella-containing groups used the same outcome definition, observation window, and safety population. The five records marked ‡ were reported as percentages without an exact numerator; displayed study-level values were estimated from the published rounded percentages and available denominators and are retained for descriptive presentation only. Individual symptom counts were not summed, because the same child may experience more than one event. Dose 1 and Dose 2 are shown separately when reported; events across doses were not added. Senders 2018 and Huang 2013 dose-specific rows include repeated participants and are not independent. These rates were not quantitatively pooled and should not be interpreted as evidence of differences in safety by vaccine type, dose, or geographic region. Citation numbers follow the main-text Vancouver scheme [11]–[53]. Range of study-level rates: approximately 28.0% to 87.3%.

S9.2 Fever Incidence After Varicella-containing Vaccination

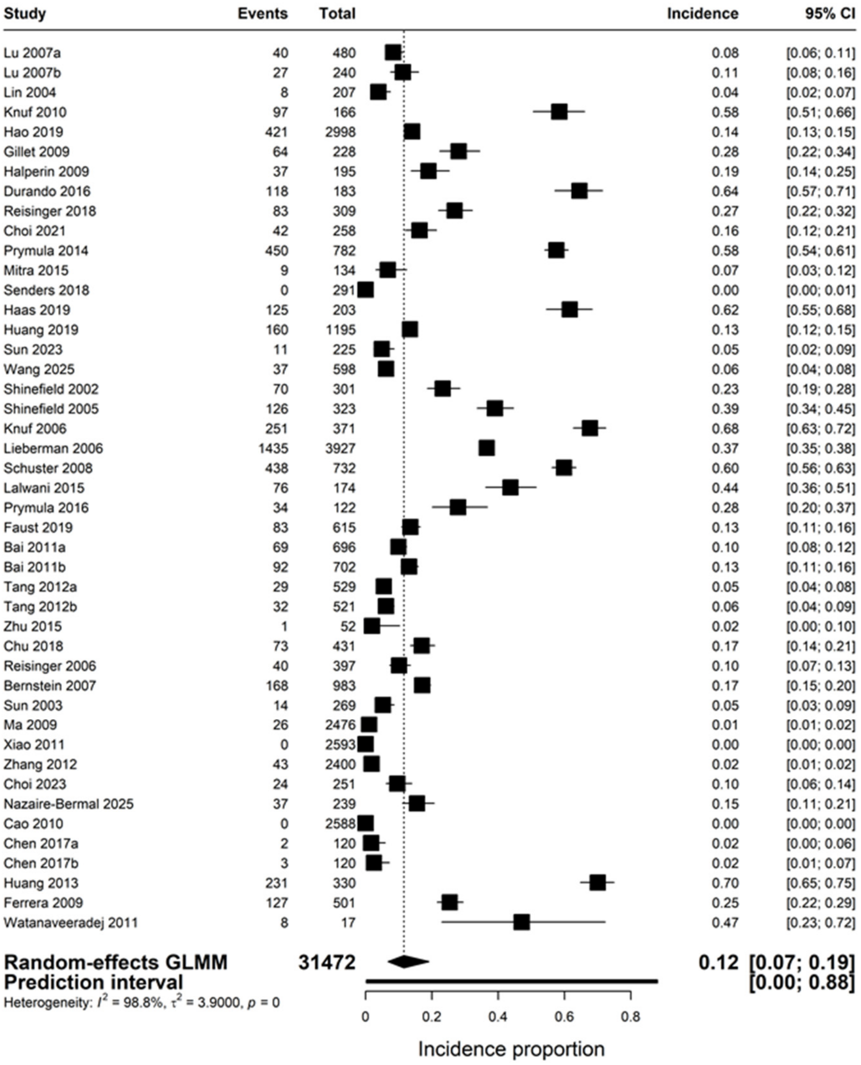

S9.3 Incidence of Adverse Events Following Monovalent Varicella and MMRV Vaccination

Adverse event	Vaccine type	Study groups	Events / participants	Pooled incidence, %	95% CI, %	I², %	P between
Fever	Monovalent varicella vaccine	28	1,530/21,551	5.287	2.615–10.396	96.0	<0.001
	MMRV	14	3,656/8,994	43.436	32.072–55.535	98.5	
Redness/erythema	Monovalent varicella vaccine	20	489/14,111	0.692	0.156–3.009	96.8	<0.001
	MMRV	14	1,697/8,994	16.502	9.797–26.451	94.9	
Pain	Monovalent varicella vaccine	24	406/16,559	0.907	0.264–3.070	96.2	<0.001
	MMRV	13	1,804/8,811	12.268	6.799–21.139	96.8	
Swelling	Monovalent varicella vaccine	25	145/17,801	0.301	0.089–1.016	91.2	<0.001
	MMRV	13	756/8,811	7.016	4.479–10.828	88.1	
Cough	Monovalent varicella vaccine	7	254/6,053	1.242	0.104–13.159	99.1	0.212
	MMRV	3	41/701	5.849	4.335–7.848	0.0	
Generalized rash	Monovalent varicella vaccine	19	332/12,666	0.308	0.066–1.413	97.6	0.551
	MMRV	14	272/8,994	0.555	0.167–1.824	89.3	
Irritability	Monovalent varicella vaccine	13	44/6,196	0.022	0.001–0.709	47.5	0.426
	MMRV	12	642/8,395	0.156	0.005–4.310	96.1	
Gastrointestinal symptoms	Monovalent varicella vaccine	23	253/17,732	0.103	0.019–0.557	96.5	0.503
	MMRV	11	587/8,200	0.020	0.000–1.738	35.7	
Injection-site rash	Monovalent varicella vaccine	17	18/10,595	0.077	0.018–0.341	0.0	0.529
	MMRV	10	9/8,017	0.028	0.002–0.470	0.0	
Fatigue	Monovalent varicella vaccine	18	304/14,307	0.062	0.007–0.527	97.1	1.000
	MMRV	9	0/7,694	0	0.000–0.048	NA	

Notes: MMRV, measles–mumps–rubella–varicella vaccine; CI, confidence interval; I², I-squared statistic. Estimates used the prespecified random-effects GLMM (meta::metaprop, sm=PLOGIT, method=GLMM, method.tau=ML) and the same outcome definitions and observation windows as the main analysis. Lin 2004 and Lu 2007 (vaccine type not reported) were excluded. The vaccine-type comparison is based on independent study groups across trials, not a within-trial head-to-head contrast of MMRV with measles–mumps–rubella vaccine plus separately administered monovalent varicella vaccine; higher or lower estimates should

not be interpreted as causal effects of vaccine type. P between is the random-effects test for subgroup differences and is shown on the first row of each outcome. Outcomes are presented separately because the same child may experience more than one adverse event. All-zero subgroups are reported as 0 with Clopper–Pearson 95% CIs. Rows are ordered from highest to lowest subgroup-specific pooled incidence.

* Pooled incidence is the inverse logit of the mean study-specific logit from the random-effects GLMM.

S9.4 Adverse Events by Vaccination Dose

Adverse event	Dose	Study groups	Events/participants	Pooled incidence, %	95% CI, %	I², %	τ²
Fever	Dose 2	5	664/1,929	26.583	13.551–45.546	98.8	0.8911
Fever	Dose 1	37	4,522/28,616	10.462	5.444–19.165	98.8	4.6384
Pain	Dose 2	5	311/1,929	9.697	1.921–37.054	97.9	3.6062
Redness/erythema	Dose 2	5	416/1,929	8.783	1.047–46.691	90.5	5.8088
Cough	Dose 2	1	10/195	5.128	2.486–9.229	NA	NA
Swelling	Dose 2	5	190/1,929	4.958	0.837–24.376	87.3	3.8692
Redness/erythema	Dose 1	29	1,770/21,176	2.461	0.867–6.784	96.2	7.3099
Cough	Dose 1	9	285/6,559	2.006	0.340–10.938	98.9	6.6771
Pain	Dose 1	32	1,899/23,441	1.937	0.740–4.978	96.9	6.8503
Swelling	Dose 1	33	711/24,683	0.786	0.314–1.954	90.5	5.7741
Generalized rash	Dose 2	4	14/1,498	0.730	0.255–2.068	0.0	0.4104
Generalized rash	Dose 1	29	590/20,162	0.375	0.120–1.169	97.5	7.3866
Febrile seizure	Dose 2	4	3/1,498	0.184	0.023–1.434	0.0	0.0934
Gastrointestinal symptoms	Dose 1	30	840/24,198	0.114	0.023–0.552	95.5	11.3973
Injection-site rash	Dose 1	24	27/17,309	0.072	0.020–0.257	0.0	3.9994
Irritability	Dose 1	21	675/13,093	0.070	0.069–0.070	92.8	19.5249
Febrile seizure	Dose 1	25	18/19,056	0.029	0.006–0.148	0.0	2.8031
Fatigue	Dose 1	24	304/20,698	0.020	0.002–0.210	96.1	13.6958
Irritability	Dose 2	4	11/1,498	0.016	0.000–22.364	0.0	16.4432
Immune thrombocytopenic purpura	Dose 1	19	1/15,138	0.003	0.000–0.909	0.0	2.3742
Anaphylaxis	Dose 1	24	4/21,322	0.001	0.000–0.859	0.0	7.6069
Gastrointestinal symptoms	Dose 2	4	0/1,734	0	0.000–0.213	NA	NA
Injection-site rash	Dose 2	3	0/1,303	0	0.000–0.283	NA	NA
Fatigue	Dose 2	3	0/1,303	0	0.000–0.283	NA	NA
Anaphylaxis	Dose 2	3	0/1,303	0	0.000–0.283	NA	NA
Immune thrombocytopenic purpura	Dose 2	3	0/1,303	0	0.000–0.283	NA	NA
Encephalitis	Dose 1	19	0/15,138	0	0.000–0.024	NA	NA
Encephalitis	Dose 2	3	0/1,303	0	0.000–0.283	NA	NA

Notes: Dose 1 and Dose 2 are dose-specific post-vaccination outcomes and are not cumulative. Knuf 2010, Prymula 2014 and Huang 2013 were classified as Dose 1 after full-text verification because the extracted numerators corresponded to post-dose-1 observation windows. Analyses were therefore restricted to Dose 1 and Dose 2. k=1 rows use Clopper–Pearson exact 95% CIs without random-effects pooling. All-zero subgroups are reported as 0 with Clopper–Pearson 95% CIs on the pooled denominator. Lin 2004 and Lu 2007 (dose not reported) were excluded. Pooled estimates: random-effects GLMM on the logit scale (meta::metaprop). Rows are ordered from highest to lowest pooled incidence. Adverse-event categories were analyzed separately and were not summed because one child may experience more than one event.

* Pooled incidence is the inverse logit of the mean study-specific logit from the random-effects GLMM.

S9.5 Exploratory Random-effects GLMM Subgroup Analyses by WHO Region

Outcome	WHO region	Arms	Pooled incidence, % (95% CI)	I^2 / τ^2	P between
Fever	WPR	21	4.38 (1.89-9.80)	98.1 / 3.8996	<0.001
	EUR	9	49.77 (38.50-61.06)	97.2 / 0.4759	
	AMR	6	13.00 (2.86-43.16)	95.9 / 3.8343	
	SEAR	4	15.71 (3.73-47.32)	94.2 / 2.3017	
	Cross-region	5	14.96 (12.52-17.77)	67.4 / 0.0323	
Pain	WPR	15	0.75 (0.23-2.36)	95.1 / 4.5382	0.001
	EUR	8	4.67 (1.65-12.52)	70.6 / 1.9121	
	AMR	6	2.74 (0.16-33.56)	95.3 / 10.6995	
	SEAR	4	13.50 (6.48-26.00)	84.8 / 0.4850	
	Cross-region	6	3.10 (0.13-43.35)	50.5 / 13.1491	
Redness/erythema	WPR	11	0.52 (0.12-2.20)	97.8 / 5.2246	0.016
	EUR	9	9.63 (2.70-29.01)	93.2 / 3.7277	
	AMR	6	2.25 (0.18-22.31)	91.1 / 8.0208	
	SEAR	4	4.39 (1.82-10.20)	0.0 / 0.2834	
	Cross-region	6	13.86 (2.77-47.59)	91.7 / 4.3696	
Swelling	WPR	16	0.19 (0.06-0.68)	87.3 / 4.4666	<0.001
	EUR	8	3.92 (1.52-9.75)	78.2 / 1.5379	
	AMR	6	1.67 (0.20-12.66)	86.5 / 5.6227	
	SEAR	4	5.41 (2.77-10.29)	0.0 / 0.1708	
	Cross-region	6	1.62 (0.12-18.54)	90.2 / 8.7431	

Abbreviations: AFR, African Region; AMR, Region of the Americas; EMR, Eastern Mediterranean Region; EUR, European Region; SEAR, South-East Asia Region; WPR, Western Pacific Region. Cross-region indicates trials conducted in at least two WHO regions. AFR and EMR had no data. P between is the random-effects subgroup Q-test. Results are exploratory between-study comparisons.

* Pooled incidence is the inverse logit of the mean study-specific logit from the random-effects GLMM.

S9.6 Rare Serious Adverse **Events** and Exact Clopper-Pearson Confidence Intervals

Outcome	Study arms	Events/participants	Incidence, %	Exact 95% CI, %
Febrile seizure	31	21/21,241	0.09887	0.06121-0.15109
Anaphylaxis	29	8/23,312	0.03432	0.01482-0.06761
Immune thrombocytopenic purpura	24	1/17,128	0.00584	0.00015-0.03253
Encephalitis	24	0/17,128	0.00000	0.00000-0.02153

S9.7. Risk-of-bias Summary Across the 43 Included Trials

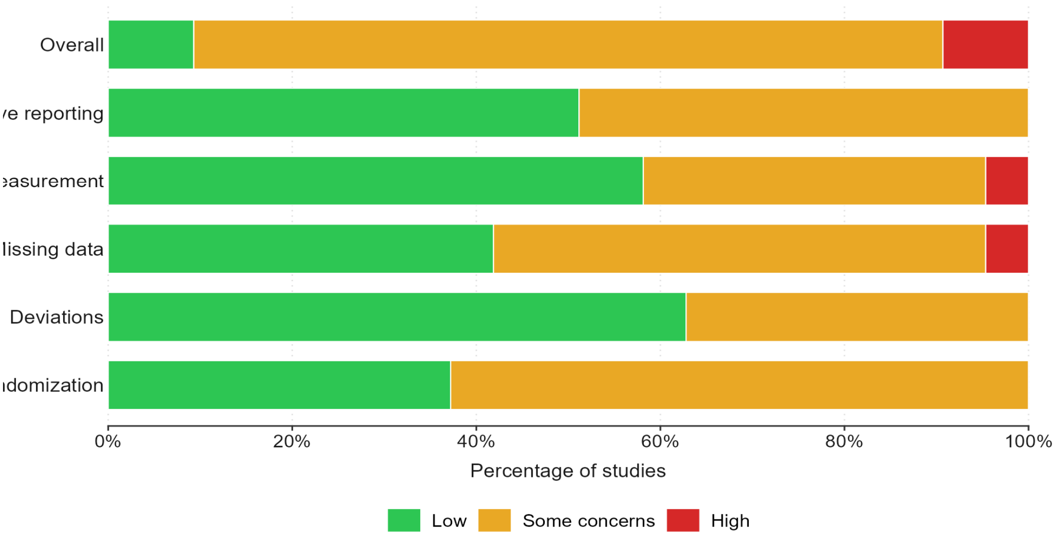

Green, low risk; yellow, some concerns; red, high risk.

S9.8 Sensitivity Analyses for Fever Incidence

Analysis	Study arms	Events/participants	Pooled incidence*, %	95% CI, %	I^2, %	τ^2	95% prediction interval, %
Main analysis	45	5,261/31,472	11.58	6.79-19.07	98.8	3.9000	0.23-88.00
Excluding age-boundary study (Knuf[27])	44	5,164/31,306	11.03	6.43-18.30	98.8	3.8585	0.22-87.22
Excluding four studies at high overall risk of bias	41	5,186/30,254	13.13	7.65-21.63	98.9	3.7542	0.29-88.85

*** Pooled incidence is the inverse logit of the mean study-specific logit from the random-effects GLMM.**

S9.9 Exploratory assessment of small-study effects

S9.9a Funnel plot of fever incidence. Study-group logits versus standard error; the vertical line is the random-effects GLMM pooled estimate.

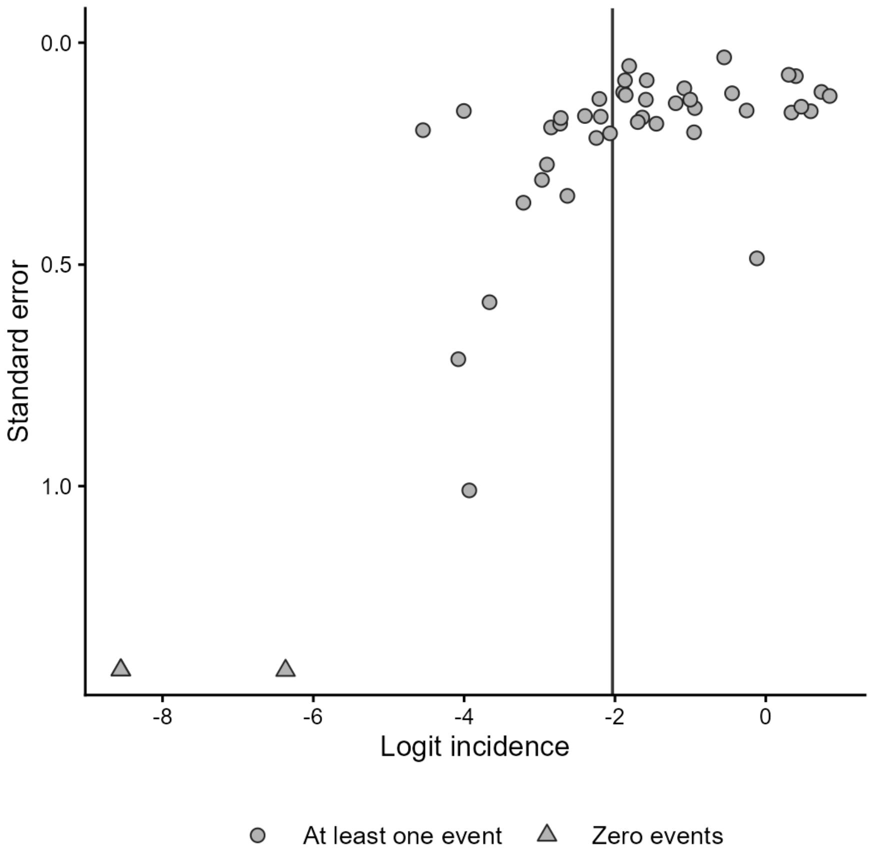

S9.9b Egger tests of funnel-plot asymmetry for common adverse events

Outcome	Study groups	Zero-event groups	Egger P
Fever	45	3	0.008
Redness/erythema	36	8	0.001
Cough	10	2	0.062
Pain	39	9	<0.001
Swelling	40	13	<0.001
Generalized rash	36	12	<0.001
Gastrointestinal symptoms	36	22	<0.001
Injection-site rash	29	19	<0.001
Irritability	27	18	0.004
Fatigue	29	21	<0.001

Notes: The vertical line in 9.9a marks the random-effects GLMM pooled logit for fever (Table 2). Triangles denote zero-event study groups. Egger's linear regression test of funnel-plot asymmetry in 9.9b used the same metaprop GLMM objects as Table 2 (meta:metabias, method.bias="linreg"). Outcomes with fewer than 10 study groups were not tested. These analyses were exploratory and were not interpreted as evidence of publication bias.
